# Modulation of RuO_2_ Nanocrystals with Facile Annealing Method for Enhancing the Electrocatalytic Activity on Overall Water Splitting in Acid Solution

**DOI:** 10.1002/advs.202409249

**Published:** 2025-01-15

**Authors:** Kangjin Song, Feng Bao, Zheling Wang, Shengding Chang, Na Yao, Haiqing Ma, Yadong Li, Caizhen Zhu, Hong Xia, Fushen Lu, Yibing Song, Jin Wang, Muwei Ji

**Affiliations:** ^1^ Key (Guangdong‐Hong Kong Joint) Laboratory for Preparation and Application of Ordered Structural Materials of Guangdong Province College of Chemical and Chemical Engineering Shantou University Shantou 515041 P. R. China; ^2^ College of Chemical and Environment Engineering Shenzhen University Shenzhen 518060 P. R. China; ^3^ Tsinghua Shenzhen International Graduate School Tsinghua University Shenzhen 518055 China; ^4^ State Key Laboratory of New Textile Materials and Advanced Processing Technologies Wuhan Textile University Wuhan Hubei 430073 P. R. China; ^5^ Key Laboratory of Functional Molecular Solids Ministry of Education College of Chemistry and Materials Science Anhui Normal University Wuhu 241002 China; ^6^ College of Materials Science and Engineering Shenzhen University Shenzhen 518071 China

**Keywords:** hydroxyl, lattice oxygen, modulation by facile annealing, RuO_2_ catalysts, stability in acid solution, water electrolysis

## Abstract

RuO_2_‐based materials are considered an important kind of electrocatalysts on oxygen evolution reaction and water electrolysis, but the reported discrepancies of activities exist among RuO_2_ electrocatalysts prepared via different processes. Herein, a highly efficient RuO_2_ catalysts via a facile hydrolysis‐annealing approach is reported for water electrolysis. The RuO_2_ catalyst dealt with at 200 °C (RuO_2_‐200) performs the highest activities on both oxygen evolution reaction (OER) and hydrogen evolution reaction (HER) in acid with overpotentials of 200 mV for OER and 66 mV for HER to reach a current density of 100 mA cm^−2^ as well as stable operation for100 h. The high‐resolution transmission electron microscopy (HRTEM) and X‐ray photoelectron spectroscopy (XPS) characterizations show that the activities of as‐prepared RuO_2_ rely on the hydroxide group/lattice oxygen (OH^−^/O^2−^) ratio, size, and crystalline of RuO_2_. The density functional theory (DFT) calculation also reveal that the OH^−^ would enhance the activities of RuO_2_ for HER and OER via modifying the electronic structure to facilitate intermediate adsorption, thereby reducing the energy barrier of the rate‐determining step. The water electrolysis by using RuO_2_‐200 as the catalyst on both anode and cathode demonstrates a stable generation of hydrogen and oxygen with high Faradic efficiency at a current density of ≈30 mA cm^−2^ and a potential of below 1.47 V.

## Graphical abstract

RuO_2_ nanoparticles prepared via a facile hydrolysis‐annealing approach are tailored hydroxide group/lattice oxygen (OH^‐^/O^2‐^) ratio, size, and crystalline for the high performance on water electrolysis in acid. The presence of OH is important to facilitate intermediate adsorption on Ru sites, and to reduce the energy barrier of the rate‐determining step.

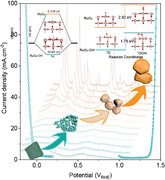

## 1 Introduction

Production of green hydrogen energy with high efficiency is regarded as an urgent issue to release the current global energy shortage and serious environmental pollution issues.^[^ ^1^, ^2^, ^3^, ^4^ ^]^ Among green technologies for energy storages, electrocatalysis on water splitting is an expected way for obtaining hydrogen via using sustainable and green energy.^[^ ^5^, ^6^, ^7^ ^]^ In the past decades, cheap metals‐based (such as Ni, Fe, Co, and so on) electrocatalysts have been widely used for water electrolysis in alkaline solutions^[^ ^8^, ^9^, ^10^ ^]^ while novel metal catalysts have been explored for high activity and long durability in acid due to the high corrosion during water electrolysis.^[^ ^11^ ^]^ Nevertheless, for green hydrogen production via water electrolysis, a larger challenge is the sluggish oxygen evolution reaction (OER), compared to the importance of hydrogen evolution reaction (HER).^[^ ^12^, ^13^, ^14^, ^15^ ^]^ Among the explored catalysts for OER, transition metal compounds (oxides, sulfide, hydroxide, phosphide, etc.) and their hetero‐structures could be facile prepared to boost OER in alkaline solution.^[^ ^16^ ^]^ For example, Zhou and Zeng fabricated 3D CoFePi networks with hierarchical porosity and found the high OER performance with a low overpotential of 0.315V at 10 mA cm^−2^ in 0.1 m KOH solution.^[^ ^17^ ^]^ For instance, Lee and co‐workers synthesized single‐phase SrIrO_3_ nanofibers by electrospinning‐calcination processes and found that the SrIrO_3_ nanofibers presented low overpotentials and Tafel slopes.^[^ ^18^ ^]^ Cho et al. studied the sodium‐decorated amorphous/crystalline RuO_2_ with oxygen vacancies to enhance the resistance to acid corrosions and oxidations, obtaining remarkable stability during OER.^[^ ^19^ ^]^ Furthermore, catalysts for OER in acid were also limited to obtain a higher current density and a lower overpotential.^[^ ^20^, ^21^ ^]^ So far, RuO_2_ and IrO_2_ are considered the benchmark catalysts for OER in acid electrolyte,^[^ ^22^ ^]^ which hints at large possibilities of Ru‐based or Ir‐based catalysts for acidic OER. Jaramillo et al. reported an IrO_x_/SrIrO_3_ thin film for OER in acid solution with 270–290 mV overpotential at 10 mA cm^−2^_geo_ for 30 h in 0.5 mol L^−1^ H_2_SO_4_ solution.^[^ ^23^ ^]^ In our previous works, the RuO_2_ cluster obtained by electrochemical leaching Sr^2+^ from SrRuO_3_ ceramic was found to perform high activity and stability on OER due to the enhanced from enlarged Jahn‐Taller distortions.^[^ ^24^ ^]^ Yang et al. reported a Ni–Co co‐doped RuO_2_ to enhance the OER activity in 0.1 m HClO_4_ solution and their results showed that the current density on RuO_2_ was over 25 mA cm^−1^ at 1.7 V_RHE_ with 64 mV dec^−1^ of Tafel slope.^[^ ^25^ ^]^ Zhang et al. optimized the 15% of Mo‐doping into mesoporous RuO_2_ with excellent properties superior to the commercial RuO_2_.^[^ ^26^ ^]^ The reported Mo‐RuO_2_ was discovered that the d‐band center of RuO_2_ moved away from the Fermi level and weakened the chemical bonds between RuO_2_ active sites and the adsorbed oxygen intermediates.^[^ ^26^ ^]^ Lin et al. prepared a Cr_0.6_Ru_0.4_O_2_ and found the stability related to the lower occupation at the Fermi level.^[^ ^27^ ^]^ Recently, a 2D ruthenium‐iridium oxide was reported with high activity on OER and an overpotential of 297 mV is required to reach a current density of 10 mA cm^−2^ with 108 mV dec^−1^ of Tafel slope.^[^ ^22^ ^]^ Qin et al. found that electronic structure and lattice strain dual engineering would enhance the OER on RuO_2_ in acid solution.^[^ ^28^ ^]^ In addition, Kuo et al. investigated the electro‐adsorption of OH and O on RuO_2_ (110) and found that the electro‐adsorption energies depend on pH and obey the scaling relation which can be reproduced by DFT simulation.^[^ ^29^ ^]^ The reported results presented that the controlled RuO_2_ performed an excellent activity on acidic OER with facile tailoring path, hinting at the emergency and possibility of obtaining RuO_2_‐based catalysts.

However, there were also some studies reporting the OER of RuO_2_ with poor performance. In addition, several OER mechanisms were investigated on RuO_2_ which hinted that the structures of RuO_2_ would result in a huge difference in the OER activity. For example, Imanishi et al. reported amorphous RuO_2_ performed higher activity than that of the crystalline RuO_2_.^[^ ^30^ ^]^ Zagalskaya and Alexandrov studied the role of defects in the interaction between adsorbate and lattice oxygen in RuO_2_ on OER and they demonstrated the lattice oxygen indeed competes with the adsorbate mechanism of OER.^[^ ^31^ ^]^ The discrepancies in the reported works hint that the microstructure of RuO_2_ catalysts influences their OER performance in acid solution and the discovery of the factors would provide important information on the design and synthesis of RuO*_x_*‐based catalysts for OER. Hence, the modulation engineering of RuO_2_ for water electrolysis also attracted lots of attention and a series of electrocatalytic sites or structures were constructed.^[^ ^32^ ^]^ For instance, cations doping to modulate the Ru/RuO_2_ heterostructure would promote the activity.^[^ ^33^ ^]^ Meanwhile, modulating RuO_2_ size not only significantly improves atomic utilization and the exposure of active sites,^[^ ^33^, ^34^ ^]^ but also promotes surface reconstruction during reaction and then accelerates the dynamics.^[^ ^35^ ^]^ Therefore, a deeper understanding of the activities of RuO_2_ for water electrolysis is still a challenge. In this regard, we prepared RuO_2_ clusters and investigated their electrocatalysis on HER and OER depending on their microstructures or coordination environment.

## 2 Result and Discussion

**Figure 1:**
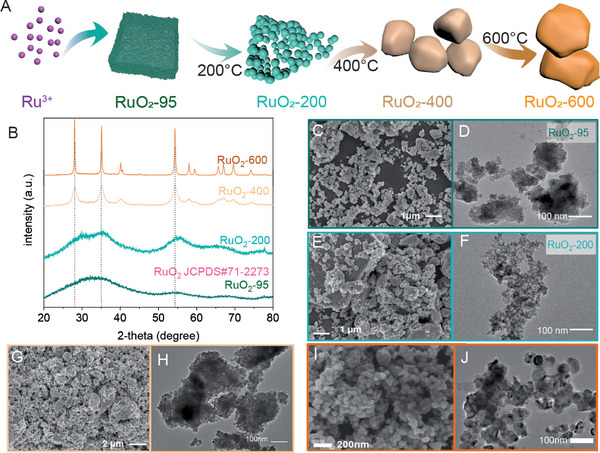
A) Schematic of the preparation of RuO_2_ nanocrystals with size controlled by temperature. B) XRD patterns of the as‐obtained RuO_2_ nanocrystals. C–J) The SEM images and TEM images of the RuO_2_ nanocrystals: C,D) RuO_2_‐95, E,F) RuO_2_‐200, G,H) RuO_2_‐400, I,J) RuO_2_‐600.

As shown in **Figure** **1**A, the RuO_2_ particles are prepared via refluxing of RuCl_3_ aqueous solution and then an annealing at a higher temperature is carried out to control their crystalline phases or sizes. The XRD patterns of the as‐prepared samples (Figure 1B) show their crystallinities depending on the annealed temperature. As shown in Figure 1B, there is a broad and weak peak ranging from 25 to 45 degrees in the XRD pattern of RuO_2_‐95, hinting at the amorphous phase or small size of samples. Two peaks in the range of 25–45 degrees and a broad peak in 50–60 degrees can be observed in the XRD pattern of RuO_2_‐200 (Figure 1B and Figure S1, Supporting Information), which can be assigned to the rutile phase of RuO_2_ (JCPDS #71‐2273). The XRD peaks of RuO_2_ become stronger because the smaller amorphous RuO_2_ particles transform into crystalline and form larger ones during the annealing at 200 °C. The process is also evidenced by the case at higher temperatures. The XRD patterns of RuO_2_‐400 and RuO_2_‐600 display the strong peaks centered at 28.0, 35.0, and 40.1 degrees, which are assigned to the peaks of facets (110), (101) and (200) of rutile RuO_2_, respectively. The XRD patterns of the samples obtained at higher temperatures display stronger and sharper peaks, illustrating that annealing brings a higher crystallinity. The synchrotron X‐ray diffraction patterns of the obtained RuO_2_ samples (Figure S2, Supporting Information) are also consistence with the XRD patterns, testifying to the high crystalline phase formed at a higher temperature. The SEM image of RuO_2_‐95 shows that the particles aggregate together to form an irregular shape (Figure 1C and Figure S3, Supporting Information). The TEM image (Figure 1D and Figure S4, Supporting Information) further confirms the small size of RuO_2‐_95 with aggregations. As the annealing temperature rises, the aggregation block of RuO_2_‐200 grows (Figure 1E and Figure S5, Supporting Information) which might be because of the loss of water molecules on the surface of RuO_2_‐95. The TEM image of RuO_2_‐200 (Figure 1F and Figure S6, Supporting Information) shows that its size is close to that of RuO_2_‐95, illustrating the aggregation of RuO_2_ particles during annealing at 200 °C. After annealing at 400 °C, some small pores on the surface of RuO_2_‐400 (Figure 1G and Figure S7, Supporting Information) can be observed, which is resulted from the growth of RuO_2_ clusters into larger particles and the aggregation of the void among the particles. As shown in the TEM images of RuO_2_‐400 (Figure 1H and Figure S8, Supporting Information), the size of the particles is about 8 nm, confirming the growth of small RuO_2_ nanocrystals into larger particles with high crystallinity. After treatment at 600 °C, the size of RuO_2_‐600 reaches about 60 nm (Figure 1I and Figure S9, Supporting Information), testifying to the rapid growth of small RuO_2_ nanoparticles at higher temperatures. The TEM images of RuO_2_‐600 (Figure 1J and Figure S10, Supporting Information) also show that the RuO_2_‐600 nanoparticles are much larger than RuO_2_‐400 and its edge is much clearer, confirming the phase transformation from atmosphere to crystalline. Combined with the XRD characterization (Figure 1B), the high crystalline phase of RuO_2_ nanoparticles can be inferred that RuO_2_‐95 clusters grow with the loss of the water molecules and phase transformation from amorphous to crystallinity during annealing.

**Figure 2:**
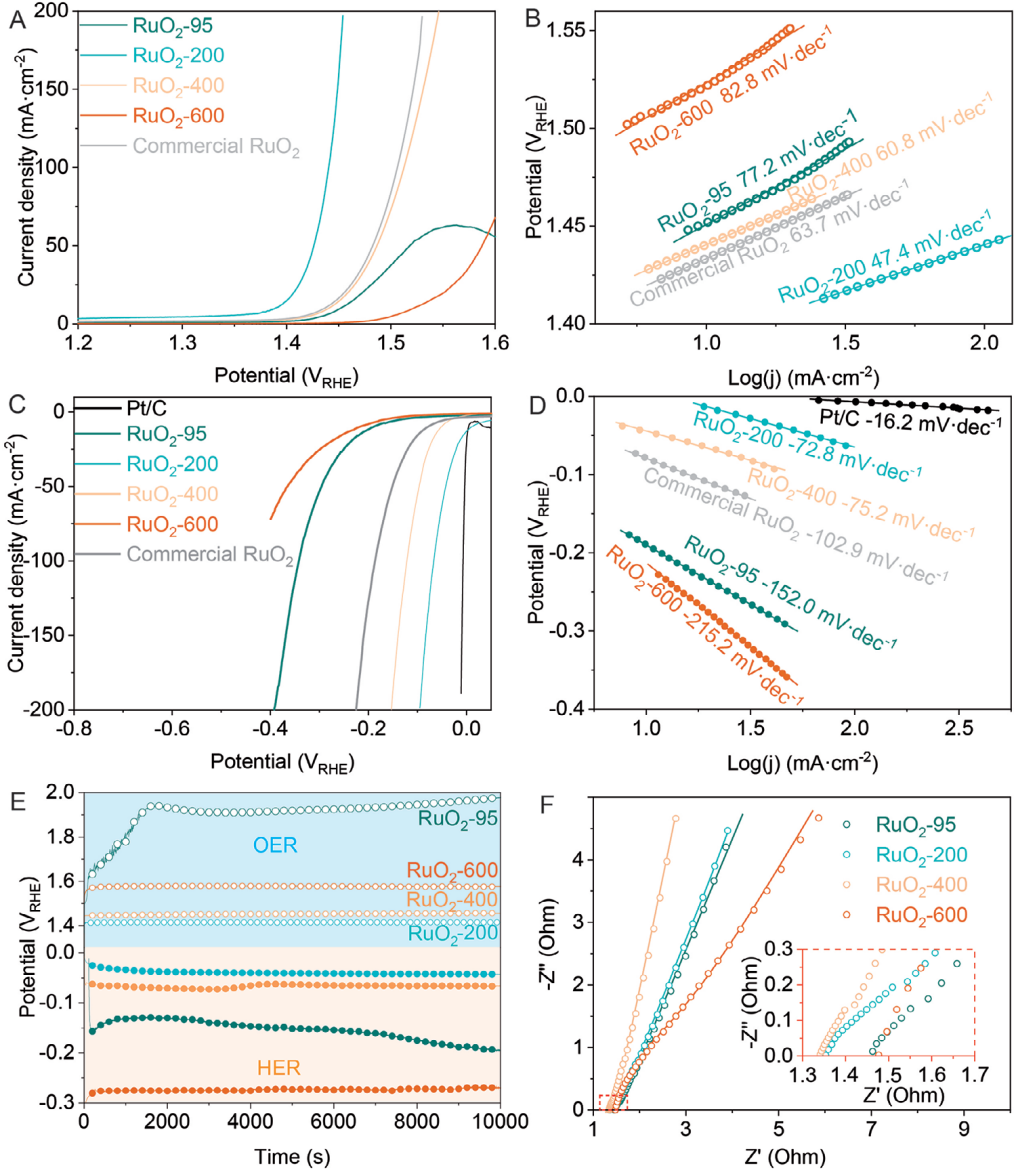
Electrocatalysis performance of series obtained samples: A,B) the polarization curves on OER and the corresponding Tafel slope. C,D) The polarization curves on OER and the corresponding Tafel slope. E) The electrochemical impedance spectra. F) The chronopotentiometry test at 10 mA cm^−2^ of current density.

To investigate the electrocatalytic activity of RuO_2_ depending on the controlled temperature, OER and HER were performed on the as‐prepared RuO_2_. As shown in **Figure** **2**A, the onset potential of OER over RuO_2_‐95 is about 1.45 V_RHE_ and the current density peaks at 63 mA cm^−2^ at 1.56 V_RHE_, forming an oxidation peak, which is attributed to the limited adsorption/desorption rate of the intermediate species. The RuO_2_‐200 and RuO_2_‐400 perform higher activities and their onset potentials are 1.39 V_RHE_ and 1.43 V_RHE_ respectively which are much lower than that of RuO_2_‐95. Besides, their current densities reach 200 mA cm^−2^ at 1.43 V_RHE_ and 1.51 V_RHE_, hinting at faster kinetics than RuO_2_‐95. In comparison, the commercial RuO_2_ was employed as OER catalysts and its performance is close to that of RuO_2_‐400. The RuO_2_‐600 performs a lower activity than the other catalysts, hinting that the larger size and better crystalline of RuO_2_ can't enhance the OER performance. Figure 2B shows that the Tafel slope on RuO_2_‐95 is 77.2 mV dec^−1^, which is a bit larger than that on RuO_2_‐400 (60.8 mV dec^−1^) or commercial RuO_2_ (63.7 mV dec^−1^) but much smaller than that on RuO_2_‐600 (82.8 mV dec^−1^), which illustrates that the large size and well crystalline of RuO_2_ never provide fast kinetics on OER. The Tafel slope of RuO_2_‐200 has the lowest value (47.4 mV dec^−1^) demonstrating the fast OER kinetics even at large current density.

For HER, RuO_2_‐95 also performs a medium activity with 200 mA cm^−2^ of current density at ‐0.4 V_RHE_ (Figure 2C). After the annealing, the activity of RuO_2_‐200 is promoted and the driven potential positive shift to ‐0.1 V_RHE_ to reach 200 mA cm^−2^ of current density. Similar to the case in the OER test, RuO_2_‐600 also performs the poorest activity on HER. The Tafel slopes on RuO_2_‐95 and RuO_2_‐600 are measured as 152.0 and 215.2 mV dec^−1^ (Figure 2D), which decade the HER via the Volmer/Tafel process. The Tafel slopes on RuO_2_‐200 and RuO_2_‐400 are calculated as 72.8 and 75.2 mV dec^−1^ (Figure 2D), illustrating the Heyrovsky/Tafel process. The HER on the RuO_2_ catalysts via different reaction processes demonstrates their difference in catalytic sides relative to their structures. Furthermore, the exchanged current densities for OER and HER on RuO_2_‐200 were calculated 3.63 and 12.59 mA cm^−2^ respectively (Figures S11 and S12, Supporting Information), which are higher than that of other catalysts and hint at the faster kinetics both on HER and OER.^[^ ^36^ ^]^ Furthermore, the electrochemical impedance spectra of RuO_2_ (Figure 2E) demonstrate that all RuO_2_ catalysts have low contact resistance and charge‐transferring resistance. As shown in Figures S13 and S14 (Supporting Information), as the driven potential enlarges, the ‐Z’’ in OER or HER decreases quickly, which is viewed as the accelerating kinetics of the reactions.^[^ ^37^, ^38^ ^]^ In the bode plots, the phase angle decreases in the low‐frequency range as the potential increases (Figure S13, Supporting Information), which manifests that the adsorbed species in the double electronic layers are transformed and hence the electric capacity on the electrode surface decreases. In the middle‐frequency range, the phase angles are down to 0 indicating a low capacitance resistance and a low charge transfer resistance.^[^ ^38^ ^]^ In the case of HER, the phase angle at the low‐frequency range also decreases when the potential shifts to ‐0.24 V_RHE_ from 0 V_RHE_, however, the peaks at the middle‐frequency range remain as the potential shift (Figure S14, Supporting Information). Hence, it is considered that the peak at low‐frequency range is attributed to the HER and that middle‐frequency range is attributed to the double layers formed by adsorbed water molecules.

At the 10 mA cm^−2^ of current density, the chronopotentiometry was carried out to characterize the stability of RuO_2_ catalysts. As presented in Figure 2F, RuO_2_‐95 is not stable enough for OER and the potential rises to 1.9 V_RHE_ in 2000 s. The other RuO_2_ catalysts perform high stability during a 10 000 s period and the potential on RuO_2_‐200 is the lowest among them. Interestingly, the RuO_2_‐200 catalyst remains stable and the potential increases only about 9 mV during 30 h operation (Figure S15, Supporting Information). For HER, the potential of RuO_2_‐200 is the lowest among the as‐prepared RuO_2_ catalysts and rises slowly during the test (Figure 2F). The Faradic efficiencies of cathode and anode reach 96.60% for HER (Figure S16, Supporting Information) with a generated rate of 0.18 mmol h^−1^ and 97.06% for OER with a generated rate of 0.09 mmol h^−1^ (Figure S17, Supporting Information), respectively.

**Figure 3:**
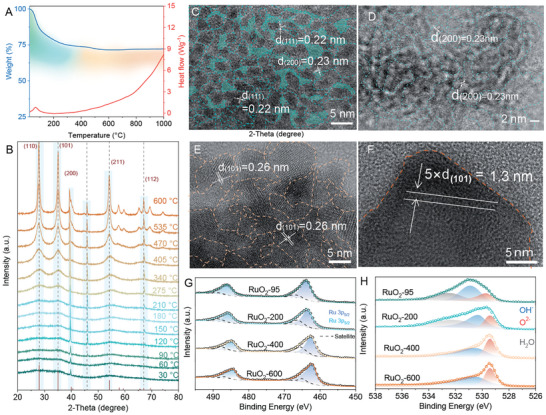
A) The TG‐DSC curve of RuO_2_. B) The in situ XRD patterns of RuO_2_ from room temperature to 600 °C. C–F) The HRTEM images of the RuO_2_ obtained from and after heat‐treatment: C) RuO_2_ ‐95, D) RuO_2_‐200, E) RuO_2_‐400, F) RuO_2_‐600. The high‐resolution XPS of G) Ru 3p and H) O 1s.

To further investigate the structure varies of RuO_2_ during annealing, the thermogravimetry (TG) and the in situ XRD tests were carried out. As shown in **Figure** **3**A, RuO_2_‐95 loses its 20.7% weight before 200 °C which is viewed as the removal of adsorbed water molecules. The corresponding DSC curve also shows that a major endothermic peak centered at 95 °C ranges from room temperature to 200 °C, which is characteristic of loss of water in RuO_2_ prepared via wet‐chemical methods. As the annealing temperature increases, the weight loss decreases slowly and remains at a plateau of 71.6% after 600 °C. An endothermic heat flow is observed from 200 °C to 1000 °C, hinting at the removal of hydroxyl groups in RuO_2_ and the RuO_2_ growth into a higher crystallinity phase. The in situ XRD patterns at the temperature range from 30 to 600 °C (Figure 3B) show that the amorphous RuO_2_‐95 transforms into crystalline RuO_2_ as the temperature rises. Figure 3B illustrates that the weak peaks of facet (200) and facet (111) are observed from 30 to 90 °C. As the temperature rises to 120 °C, the XRD peak intensity of facet (210) increases and the peaks become strong and sharp in a higher temperature range between 150 °C and 210 °C. Meanwhile, the facet (112) peak appears, also confirming the formation of a high crystalline phase. Besides, in the temperature range from 90 °C to 210 °C, the amorphous peak from 25° to 40° divides into two peaks centered at about 28° and 35° which are assigned to the facet (110) and facet (101) (Figure S18, Supporting Information). It manifests that RuO_2_‐95 loses water molecules and grows into larger nanoparticles during the heat treatment at 200 °C. As the test temperature rises, the intensities of each peak increase and become sharper, illustrating the growth of RuO_2_ as the temperature rises. Figure 3C and Figure S19 (Supporting Information) present the HRTEM images of RuO_2_‐95 with a size of less than 2 nm and the lattice fringe distances are measured as 0.23 nm and 0.22 nm that are assigned to the facets (200) and (111) of RuO_2_. Besides, lots of interfaces (labeled as green dash lines in Figure 3C) and amorphous phase zones (labeled as green covers in Figure 3C) are found in RuO_2_‐95 particles, which hints at the low crystallinity resulting from the small size and presence of water molecules. The HRTEM images of RuO_2_‐200 (Figure 3D and Figure S20, Supporting Information) show that the crystallinity becomes higher after annealing at 200 °C. Although there also exist lots of interfaces between the lattices, the amorphous phase zones transform into crystalline zones, indicating better crystallinity than that of RuO_2_‐95. The crystalline phase of the as‐prepared RuO_2_ depending on the temperature can also be testified by the HRTEM images of RuO_2_‐400 (Figure 3E and Figure S21, Supporting Information) and RuO_2_‐600 (Figure 3F and Figure S22, Supporting Information). Figure 3E shows that the lattice interface in RuO_2_‐400 is much less than that in RuO_2_‐200 or RuO_2_‐95 and the corresponding crystalline zone also becomes larger. As displayed in Figure 3F, the lattice fringe distance of RuO_2_‐600 is measured at 0.26 nm, which is assigned to the facet (101). Furthermore, the monocrystalline is observed in the HRTEM images of RuO_2_‐600 particles (Figure 3F and Figure S22, Supporting Information). The in situ XRD and HRTEM characterizations confirm that the RuO_2_ obtained by wet‐chemical methods processes water molecule loss and crystalline phase increase as the temperature rises.

What should be noted, as shown in Figure 2A,C, the capacities for OER and HER on the RuO_2_ are incompletely dependent on their sizes or interfaces. As reported in our previous work,^[^ ^24^ ^]^ the RuO_2_ clusters with a size of about 2 nm perform a high activity and stability in acid solution, which resulted from its enhanced Jahn‐Teller distortion of Ru–O octahedral. Hence, the growth of RuO_2_ nanoparticles reduces the Jahn Teller distortion and then reduces the activity. In this case, the RuO_2_ catalyst with a smaller size performs a higher activity and consequently, annealing treatment is an effective way to modulate their activities. Nevertheless, the size of RuO_2_‐95 is close to that of RuO_2_‐200 but brings a poorer performance, which hints that the size of RuO_2_ is not the only reason influencing the activity. To further reveal the difference in the chemical state of Ru and O in our RuO_2_ samples, the XPS technique was employed to provide detailed information. As shown in the survey XPS of the RuO_2_ catalysts (Figure S23, Supporting Information), the Ru 3d and 3p peaks are located at 280 and 463 eV respectively while O 1s peaks are located at 531 eV. The high‐resolution XPS of Ru 3d (Figure S24, Supporting Information) in RuO_2_‐95 illustrates the peaks centered at 281.2 and 284.3 eV with board peak width, which is assigned into Ru 3d_3/2_ and 3d_5/2_ of Ru(IV) respectively. The Ru 3d_3/2_ and 3d_5/2_ peaks in the RuO_2_‐200 center at 281.1 and 285.2 eV and a positive shift occurs in comparison with that of the RuO_2_‐95 (Figure S24 and Table S1, Supporting Information). The negative shift also can be observed in the Ru 3d XPS of RuO_2_‐400 (280.9 and 285.1 eV) and RuO_2_‐600 (280.8 and 284.9 eV). The peaks at around 283.5 and 286.1 eV in RuO_2_‐95 and RuO_2_‐200 might result from the Ru atoms on the surface, dedicating that the small size and OH^−^ groups on the surface would modulate the electronic structure of the Ru center. This location shift and shape‐changings of XPS peaks also occur in the Ru 3p XPS of RuO_2_ as the annealing temperature rises. As shown in Figure 3G, the Ru 3p_1/2_ and 3p_3/2_ peaks of RuO_2_‐95 are at 462.9 and 484.9 eV. What should be noted, the peaks negative shift and become narrow in the RuO_2_ samples dealt with higher temperature (Table S1, Supporting Information). Compared to the XPS of Ru, the XPS changes and shifts of O 1s are more obvious. The O 1s peak of RuO_2_‐95 (Figure 3H) exhibits a broadened and asymmetric profile and can be deconvoluted into O 1s components of the free water (532.5 eV), hydroxyl groups (OH^−^, 531.3 eV), and lattice oxygen (O^2−^, 529.6 eV) (Table S2, Supporting Information).^[^ ^25^ ^]^ In addition, the area integral intensity ratios of OH^−^/O^2−^ in the series of RuO_2_ are summarized in Table S3 (Supporting Information) and clearly demonstrate the changes in the OH^−^ and the O^2−^ during the heat treatment. As listed in Table S3 (Supporting Information), the ratio of OH^−^/O^2−^ in RuO_2_‐95 is the smallest due to the large number of water molecules on its surface while it increases after heat treatment, which is considered as the formation OH^−^ and removal of water. Besides, as continue growth of small RuO_2_ into larger nanoparticles at high temperatures, the OH^−^ also is removed and forms a new lattice O^2−^, changing the ratio of OH^−^/O^2−^ and the activity sites.

**Figure 4:**
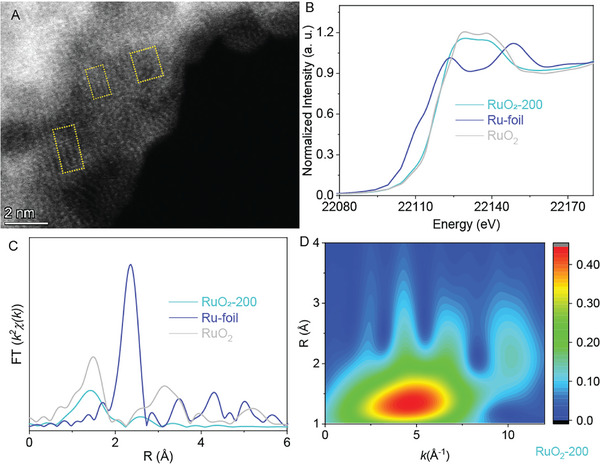
A) The spherical aberration‐corrected high angle annular dark‐field scanning TEM images and the B–D) X‐ray absorption analysis of RuO_2_‐200: B) The normalized Ru K‐edge XANXS spectra of the RuO_2_‐200, Ru foil, and RuO_2_. C) The Fourier transform‐EXFAS of the RuO_2_‐200, Ru foil and RuO_2_. D) The wavelet transform‐EXAFS of the RuO_2_‐200.

To further reveal the structure of RuO_2_‐200, the spherical aberration TEM and X‐ray absorption spectroscopy (XAS) were used for the characterizations (**Figure** **4**). As illustrated in Figure 4A, the spherical aberration TEM image illustrated the lattice with short‐range order and serious distortion (in the yellow dash line zone), which was assigned to the OH group on the surface of the RuO_2_‐200. Figure 4B shows that the normalized Ru K‐edge XANXS spectrum of RuO_2_‐200 was close to that of RuO_2_, manifesting the identical chemical valence of RuO_2_‐200 and bulk RuO_2_. The Fourier transition EXAFS of the RuO_2_‐200 (Figure 4C) shows that the peak is at 1.47 Å but fades quickly as *R* increases, resulting from the small size of RuO_2_‐200. The Ru K‐edge EXAFS fitting results (Figure S25 and Table S4, Supporting Information) reveal that the coordination number of Ru in RuO_2_‐200 is 6 and the Ru–O length are 1.91 and 2.04 Å, which is coincidence with the parameters of RuO_2_ cluster and indicates the distortion.^[^ ^24^ ^]^ The wavelet transform‐EXAFS (Figure 4D and Figure S26, Supporting Information) further confirms the presence of the Ru–O bond in RuO_2_‐200 without the Ru–Ru bond.

**Figure 5:**
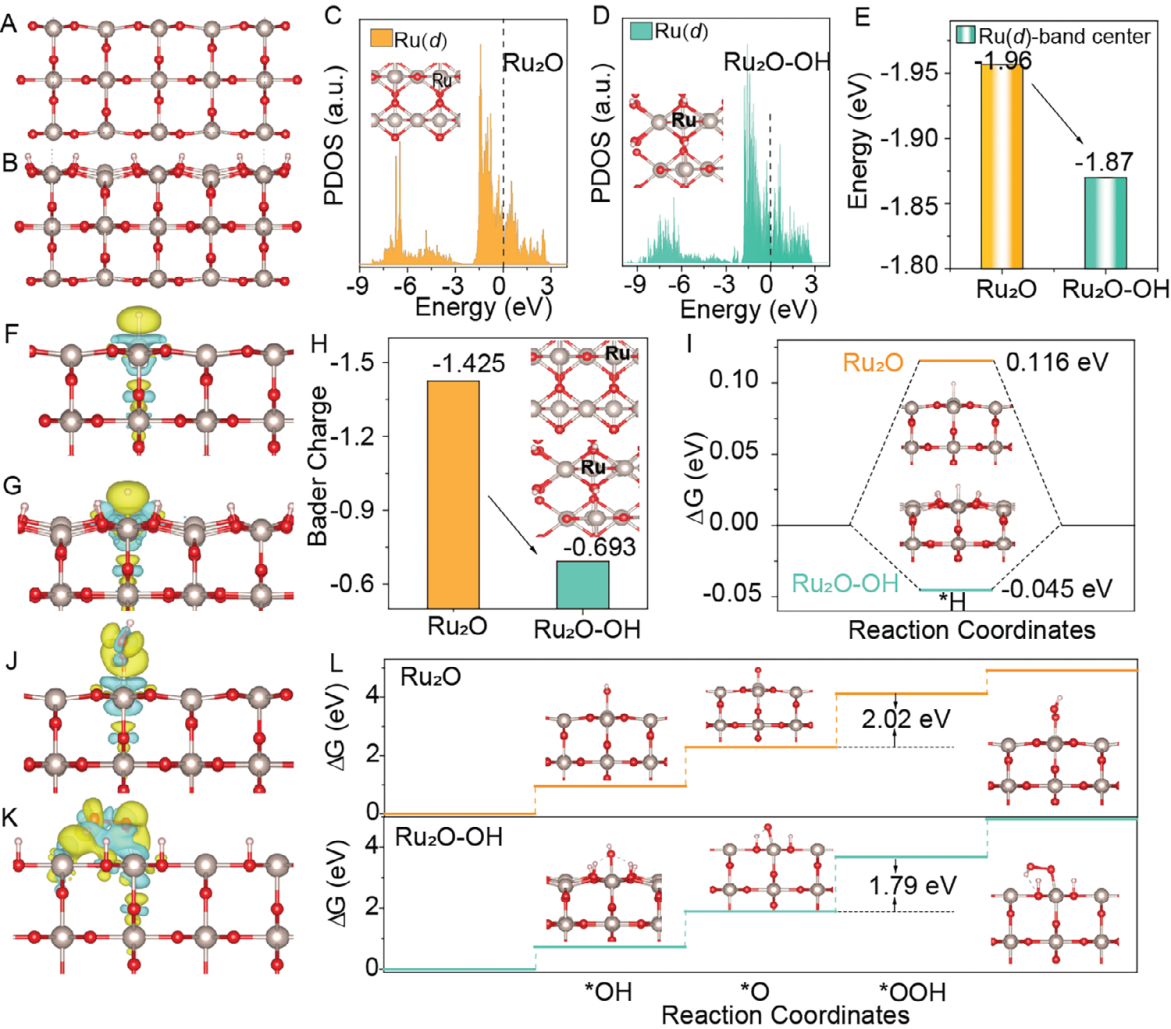
The geometric configuration of A) RuO_2_; B) RuO_2_‐OH. C) PDOS plots of Ru‐d states in RuO_2_. D) PDOS plots of Ru‐d states in RuO_2_‐OH. E) The Ru‐d band center of RuO_2_ and RuO_2_‐OH. F,G) Electronic charge difference of *H‐RuO_2_ and *H‐RuO_2_‐OH, respectively. H) The Bader charge of RuO_2_ and RuO_2_‐OH. I) Free energies of H adsorption on RuO_2_ and RuO_2_‐OH. J,K) Electronic charge difference of *OOH‐RuO_2_ and *OOH‐RuO_2_‐OH, respectively. L) Gibbs free energy illustration by RuO_2_ and RuO_2_‐OH catalysts during the OER process.

The density functional theory (DFT) calculations were carried out to further understand the mechanism of enhanced water splitting performance in acidic electrolytes by the OH group on the surface of RuO_2_‐200 and the employed theory models for RuO_2_ and RuO_2_‐OH are shown in **Figure** **5**A,B, respectively. First, the geometric configuration of *H‐RuO_2_ and *H‐RuO_2_‐OH (Figure S27, Supporting Information) were used for the calculation of the adsorption free energy of hydrogen (∆G_*H_), which is usually used for evaluating the HER performance. As reported in the previous work,^[^ ^39^ ^]^ catalysts with hydrogen adsorption free energy of close to zero (∆G_*H_ ≈ 0) are considered promising candidates for acidic HER. Figure 5C,D shows the d‐orbital partial density of states (d‐PDOS) analysis on the Ru atoms of RuO₂ and RuO₂‐OH, whose results are present in Figure 5E. The d‐band center shifted to ‐1.87 eV from ‐1.94 eV after introduction of OH (Figure 5E), illustrating the d‐band center of Ru atoms closer to the Fermi level. According to the d‐band theory, a higher of *d*‐band center would lead to stronger binding interaction between the catalysts and adsorbates.^[^ ^40^, ^41^ ^]^ Figure 5F,G and Figure S28 (Supporting Information) show that more electron cloud overlaps and the highest electronic local functions are observed in *H‐RuO_2_‐OH compared to that on *H‐RuO_2_, confirming the enhanced *H adsorption in RuO_2_‐OH. Furthermore, the Bader charge analysis for both RuO_2_ and RuO_2_‐OH were also performed and the charge of Ru atom is ‐0.693 in RuO_2_‐OH, while the charge of Ru atom in RuO_2_ is calculated to be ‐1.425 (Figure 5H). Such charge analysis results suggest that OH doped in RuO_2_ possesses a much lower valence than Ru atoms in pure RuO_2_. The Ru atoms with much negative charge will adsorb *H easier by capturing positively charged H atoms through the strong electrostatic attraction interaction, and then pave the way for subsequent reaction steps. As shown in Figure 5I, the free energy diagrams (FED) on RuO_2_ and RuO_2_‐OH were calculated and the ΔG_*H_ value (−0.045 eV) of RuO_2_‐OH is more thermo‐neutral than the undoped RuO_2_ (0.116 eV), suggesting that RuO_2_ after OH engineering would possess enhanced HER performance. Similarly, compared to RuO₂, RuO₂‐OH has a higher Ru‐d band center, indicating enhanced adsorption of the oxygen intermediate on Ru sites. Furthermore, in the calculations, the oxygen intermediates form hydrogen bonds with OH in RuO₂‐OH when adsorbed on RuO₂‐OH, which is not present in RuO₂ (Figures S29 and S30, Supporting Information). This further facilitates the adsorption of the oxygen intermediate on RuO₂‐OH, resulting in more electron cloud overlap in *OOH‐RuO_2_‐OH than that on *OOH‐RuO_2_ (Figure 5J,K) and then enhancing the *OOH adsorption in RuO_2_‐OH. As expected, the enhanced adsorption strength of *OOH caused by introducing OH in RuO_2_ is favorable to decrease the rate‐determining step (RDS) (*O→*OOH) (≈1.79 eV), which requires a much lower RDS energy than that of the RuO_2_ catalyst (≈2.02 eV) (Figure 5L). Therefore, the excellent water splitting performance of RuO₂‐200 originates from the presence of OH which modifies the electronic structure and facilitates H adsorption, bringing ∆G_*H_ of RuO₂‐OH close to zero and enhancing HER activity. The OH in RuO_2_‐200 also strengthens the adsorption of the oxygen intermediate on Ru sites through electronic structure modification and hydrogen bonding, thereby reducing the energy barrier of the rate‐determining step and enhancing OER activity.

**Figure 6:**
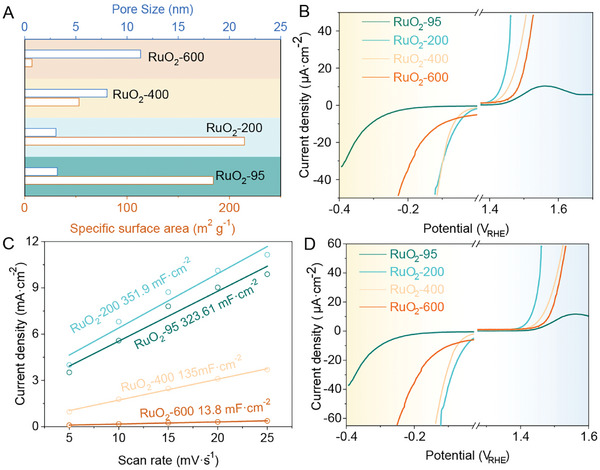
A) the specific area and pore size of the series of electrocatalysts and B) the specific activities of HER and OER depending on BET. C) The ECSA of the series of electrocatalysts and D) the corresponding activities on HER and OER.

To characterize the special activity of RuO_2_, the specific areas and the pore sizes of the series of RuO_2_ samples are measured by using the BET method (Figures S31–S34, Supporting Information) and the corresponding results are listed in **Figure** **6**A and Table S5 (Supporting Information). As shown in Figure 6A, RuO_2_‐95 contains the largest specific area with 184.1 m^2^ g^−1^. It is noticeable that although RuO_2_‐95 and RuO_2_‐200 are both RuO_2_ clusters with similar shape and size, the BET area of RuO_2_‐200 (214.6 m^2^ g^−1^) is larger than that of the RuO_2_‐95, which might be because of removal of the water molecules adsorbed on the RuO_2_ surface and production of new adsorbed sites. The pore size characterizations of the RuO_2_ catalysts also show that the pore sizes of RuO_2_‐95 and RuO_2_‐200 are smaller than 5 nm and enlarge to 10 nm after heat‐treating at 400 °C and 600 °C, which results from the smaller pores joining and form the larger pores. The specific activities of the RuO_2_ catalysts (Figure 4B) are calculated by their polarized curve and it is testified that RuO_2_‐200 performs the highest activity on both HER and OER. Besides, the electrochemical surface area (ECSA) also was carried out to reveal the reaction activity of the electrodes (Figures S35–S38, Supporting Information). The calculated areas also illustrated that *C*_dl_ reaches 351.9 mF cm^−2^ which is a little higher than that of RuO_2_‐95 and much higher than that of RuO_2_‐400 and RuO_2_‐600 (Figure 4C). The activities depending on the ECSA (Figure 4D) also reveal that RuO_2_‐200 performs the highest activity on HER and OER. Hence, the specific area and ECSA measurements also testify that RuO_2_‐200 contains the most active sizes.

**Figure 7:**
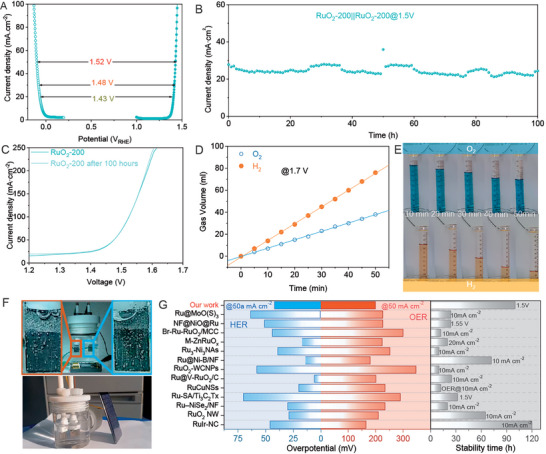
A) The polarized curves on HER and OER. B) The *i*–*t* curve of water splitting on the RuO_2_‐200 at 1.5 V. C) The polarized curve of RuO_2_‐200 on water splitting before and after 100 h of chronoamperometry test. D) Comparisons of the overpotentials at 10 mA cm^−2^ on HER/OER and stabilities of Ru‐based catalysts and RuO_2_‐200 in this work.^[^ ^34^, ^42^, ^43^, ^44^, ^45^, ^46^, ^47^, ^48^, ^49^, ^50^, ^51^, ^52^, ^53^ ^]^

Considering the excellent catalytic capability of RuO_2_‐200 on both HER and OER, the electrocatalytic overall water splitting was thus studied. From the LSV curves in a three‐electrodes system, the voltage for water‐splitting rises slowly as current density increases (**Figure** **7**A). As shown in Figure 7A and Figure S39 (Supporting Information), the voltage for overall water splitting is about 1.50 V to reach 100 mA cm^−2^ of current density while the current density enlarges to 300 mA cm^−2^, the voltage just only increases about 80 mV. The overall water splitting by using RuO_2_‐200 as both anode and cathode in a two‐electrode system was also investigated at 1.5 V of cell voltage without IR compensation. As shown in Figure 7B, the current density reaches around 30 mA cm^−2^ and remains well in the 100 h. Furthermore, the chronopotentiometry (CP) test was carried out at a series of current densities to confirm the high activity of RuO_2_‐200 on water overall splitting. As presented in Figure S40 (Supporting Information), about 1.38 V of voltage is needed to reach 10 mA cm^−2^ and the voltage remains well lower than 1.40 V during a 1 h test. To enlarge the current density to 100 mA cm^−2^ from 10 mA cm^−2^, the driven voltages are measured as 1.46 V (at 20 mA cm^−2^), 1.61 V (at 50 mA cm^−2^), and 1.78 V (at 100 mA cm^−2^), respectively. To sum up, the RuO_2_‐200 catalyst performs the prominent activity as well as the long‐term stability for overall water splitting. It should be noted that the difference between the CP results and the voltages calculated from the polarized curves is attributed to the resistance of the HClO_4_ solution. In each chronopotentiometry test period, the voltage at different current densities locals at the corresponding plateau without obvious increasement, demonstrating the good stability of RuO_2_‐200. The stability also can be testified by the comparison of the polarized curves of RuO_2_‐200 before and after on 100 h water splitting test. Figure 7C shows that the two polarized curves of RuO_2_ are very closed, manifesting the excellent stability of the RuO_2_‐200. Furthermore, the structures of RuO_2_‐200 were also checked after HER and OER tests to investigate the stability. Figures S41 and S42 (Supporting Information) present the HRTEM images of RuO_2_‐200 after the OER/HER test and indicate the remaining size and interface which ensures the activities and stabilities of RuO_2_‐200. The HAADF‐STEM images and the corresponding element mappings (Figures S43 and S44, Supporting Information) show the uniformly dispersed Ru and O elements on the samples, further confirming the well‐remaining compositions during the HER and OER test period. The XPS of Ru 3p (Figure S45, Supporting Information) demonstrates the Ru chemical state after HER or OER is very close to that of RuO_2_‐200 before the electrochemical test. Therefore, RuO_2_‐200 performs high stability for water splitting in an acid solution. Compared to the reported catalysts on HER and on OER^[^ ^34^, ^42^, ^43^, ^44^, ^45^, ^46^, ^47^, ^48^, ^49^, ^50^, ^51^, ^52^, ^53^ ^]^ (Figure 7D and Table S5, Supporting Information), the RuO_2_‐200 performs well bifunctions with high activities (the needed overpotentials to reach the current density of 50 mA cm^−2^ for HER and OER are only 42 and 199 mV, respectively.) and stability on water splitting in acid, which provides a potential way for green hydrogen production by water electrolysis.

**Figure 8:**
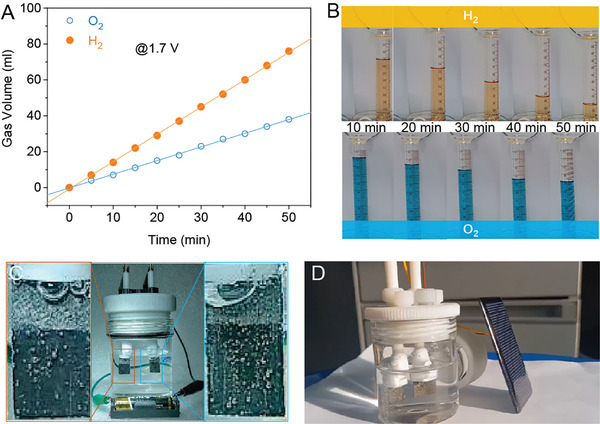
A) The gas volume of O_2_ and H_2_ produced by using the RuO_2_‐200 at 1.7 V of cell voltage and B) the corresponding photographs in different working times. C) The photographs of water splitting on the RuO_2_‐200 using Zn–Mn batteries (1.5 V) and D) a solar cell under natural irradiation.

To investigate the applications of water overall splitting, several cases were carried out by using different cells or electric devices. For the applications under 1.7 V of voltage, the produced H_2_ and O_2_ are collected and as presented in **Figure** **8**A,B, the volume ratio of the produced gas on the anode and cathode is close to 1:2. By using a commercial AA battery with 1.5 V (Figure 8C and Video S1, Supporting Information) of voltage and photo‐voltage cell (Figure 8D and Video S2, Supporting Information) driven under the natural irradiation, the abundant bubbles produced on the anode and cathode indicating the water splitting is successfully driven on the RuO_2_‐200. Moreover, the H_2_ and O_2_ bubbles during the test can be observed letting out in time from the electrode to avoid further gathering and growing up.

## 3 Conclusion

In summary, we have successfully synthesized RuO_2_ catalysts through hydrolysis‐annealing methods, which shows high activity and stability on both OER and HER. By investigating the annealing process of RuO_2_, the change of crystalline and interfaces between the small RuO_2_ nanoparticles were revealed. The more important thing is that the ratio of OH^−^/O^2−^ of RuO_2_ also varied during the annealing process, which was considered one of the dependent factors on water splitting. Besides, the excellent water splitting performance of RuO₂‐200 was further revealed by DFT calculation and the presence of OH was consider modifying the electronic structure and facilitating intermediate adsorption on Ru sites, and thereby reducing the energy barrier of the rate‐determining step.

## 4 Experimental Section

### Chemical and Reagents

RuCl_3_ (99.9%) and HClO_4_ solution (85%) were purchased from Aladdin Co. Ltd. All the chemicals were used without further purification. The water used for preparing solutions is ultrapure water whose resistance is 18.2 M ohm.

### Preparation of RuO_2_ Catalysts

RuCl_3_ (124.4 mg, 0.6 mmol) was dissolved into ultrapure water (5 mL) to form a RuCl_3_ solution with a concentration of 0.12 mol L^−1^. Then the RuCl_3_ solution (5 mL) was added to the ultrapure water (20 mL) in a 50 mL flask and then the solution was kept reflexing at 95 °C for 18 h, producing a black precipitate. After cooling to room temperature, the precipitate was collected by centrifugation (7000 rpm for 10 min), and purified by dispersing in water and centrifugation (7000 rpm for 10 min) for several times. The obtained RuO_2_ was dried in an oven at 50 °C for 3 h and marked as RuO_2_‐95. The RuO_2_‐95 was annealed at 200 °C (or 400 °C, or 600 °C) for 2 h and the obtained samples were marked as RuO_2_‐200 (or RuO_2_‐400, or RuO_2_‐600).

### Characterizations

The X‐ray diffraction (XRD) patterns were collected by using a Rigaku MiniFlex 600 diffractor with Cu Kα (*λ* = 0.154 nm) radiation at 40 kV of operated voltage and 20 mA of operated current. The scanning rate is 5 ° min^−1^ from 20° to 80°. The in situ XRD patterns were collected by using an X‐ray diffractor (PANalytical Empyrean, Britain) with Cu Kα (*λ* = 0.154 nm) as the X‐ray source and the operated voltage and current were 40 kV and 40 mA, respectively. The morphologies of the obtained samples were characterized using a scanning electron microscope (SEM) with 30 kV of accelerated voltage. The low‐resolution transmission electron microscopy (TEM) and high‐resolution transmission electron microscopy (HRTEM) images were performed on a transmission electron microscope (FEI TALOS 200X, USA) with 200 kV of accelerated voltage. The X‐ray photoelectron spectroscopy (XPS) characterization was carried out by using a Thermo K‐Alpha X‐ray photoelectron spectroscopy (Thermo Fisher Scientific) with Mono Al Kα source (1486.6 eV). The special areas and the pore sizes of the samples were obtained by using a surface area and aperture analyzer (BSD‐PS1/2, Beishide Instrument Technology Co., Ltd.) and calculated with BET methods. The thermogravimetric analysis was carried out by using a thermogravimetric analyzer (Mettle TGA 2, Mettler Toledo Co. Ltd.). The spherical aberration‐corrected high angle annular dark‐field scanning transmission electron microscopy of RuO_2_‐200 was performed on a FEI Spectra 300 with 300 kV of accelerated voltage. All XAFS data were measured at the Shanghai Synchrotron Radiation Facility (SSRF, China) under 3.5 GeV, 260 mA beam conditions using beamline BL14W1 with a Si(111) double crystal monochromator and the energy was calibrated using Fe foil. A N_2_‐filled ionization chamber was used to measure the incident flux. The size of the Synchrotron beam at the sample location was 0.3 mm(V)×0.3 mm(H) and All of data were collected in the transmission mode at room temperature.

### Electrocatalysis on Water Splitting

The electrocatalytic performance was conducted by an electrochemical workstation (CHI 760E, Chenhua Co. Ltd.) with a typical three‐electrode configuration, in which a graphite rod was taken as the count electrode and an Ag/AgCl (saturated KCl solution) electrode was used as the reference electrode. The work electrode was by loading the as‐prepared RuO_2_ catalysts onto carbon papers. Typically, RuO_2_ powder (20 mg) was dispersed in 0.4 mL of a mixed solution containing isopropanol (380 µL) and nafion solution (20 µL, 5%) via ultrasonication and then the resulted ink (100 µL) was doped onto a carbon paper at room temperature. The loading density of the RuO_2_ is 5 mg cm^−2^. During the characterization period, the HClO_4_ solution (1 mol L^−1^) was used as the electrolyte. More details were provided in the Supporting Information.

## Conflict of Interest

The authors declare no conflict of interest.

## Supporting information



Supporting Information

Supplemental Video 1

Supplemental Video 2

## Data Availability

The data that support the findings of this study are available in the supplementary material of this article.
