# Supplementary material for: Modulation of RuO2 Nanocrystals with Facile Annealing Method for Enhancing the Electrocatalytic Activity on Overall Water Splitting in Acid Solution
Source: Adv Sci (Weinh). 2025 Jan 15;12(9):2409249. doi: 10.1002/advs.202409249 (PMC11884526; doi:10.1002/advs.202409249)
Supplement: Supplementary file 1 — Supporting Information [file ADVS-12-2409249-s003.pdf]

ADVANCED SCIENCE Open Access

Supporting Information

for *Adv. Sci.*, DOI 10.1002/advs.202409249

Modulation of RuO_2 Nanocrystals with Facile Annealing Method for Enhancing the Electrocatalytic Activity on Overall Water Splitting in Acid Solution

Kangjin Song, Feng Bao, Zheling Wang, Shengding Chang, Na Yao, Haiqing Ma, Yadong Li, Caizhen Zhu, Hong Xia, Fushen Lu, Yibing Song*, Jin Wang* and Muwei Ji**

Modulation of RuO_2 nanocrystals with facile annealing method for enhancing the electrocatalytic activity on overall water splitting in acid solution

Kangjin Song[a,#], Feng Bao[b,#], Zheling Wang[a,#],Shengding Chang[c,#], Na Yao[d,*], Haiqing Ma[c], Yadong Li[b], Caizhen Zhu[b], Hong Xia[a], Fushen Lu[a], Yibing Song[a,*], Jin Wang[c,e,*], and Muwei Ji[a,*]

a. Key (Guangdong-Hong Kong Joint) Laboratory for Preparation and Application of Ordered Structural Materials of Guangdong Province, College of Chemical and Chemical Engineering, Shantou University, Shantou, 515041, P. R. China
b. College of Chemical and Environment Engineering, Shenzhen University, Shenzhen 518060, P. R. China
c. Tsinghua Shenzhen International Graduate School, Tsinghua University, Shenzhen 518055, China
d. State Key Laboratory of New Textile Materials and Advanced Processing Technologies, Wuhan Textile University, Wuhan, Hubei 430073, P. R. China.
e. Key Laboratory of Functional Molecular Solids Ministry of Education, College of Chemistry and Materials Science, Anhui Normal University, Wuhu, 241002, China

#：The authors contributed equally to this work.

Corresponding Author: yaona@wtu.edu.cn (Na Yao); ybsong@stu.edu.cn (Yibing Song);

wangjin19@szu.edu.cn (Jin Wang); mwji@stu.edu.cn (Muwei Ji).

Experiment and simulation details

ES1. Electrocatalysis characterization:

The electrocatalytic performance was conducted by an electrochemical work-station (CHI 760E, Chenhua Co. Ltd.) with a typical three-electrode configuration, in which a graphite was taken as the count electrode. An Ag/AgCl (saturated KCl solution) electrode was used as reference electrode. And the potential vs RHE was calculated by the equation as follow:

$$E_{RHE} = E + 0.059pH + 0.197.$$

The C_{dl} was obtained by collecting cyclic voltammogram curves at various scan rates of 5, 10, 15, 20, and 25 mV s^{-1} in the non-faradaic range and the electrochemical surface area (ECSA) of RuO_2 electrode was obtained from the C_{dl} value using the equation as follow:

$$ECSA=C_{dl}/C_F$$

Where, the C_F was the specific capacitance of 0.04 mF/cm^2.

ES2. Calculation of Faraday efficiency: Faradaic efficiency measurements were carried out in a two-electrode system for the overall water splitting. For H_2-measurements and O_2-measurements, the electrolytic cell was completely closed, and N_2 was blown in to drive out the residual air for 30 minutes. The hydrolysis reaction was carried out at 10 $mA{\cdot}cm^{-2}$ current density for 10800 s. then the gas chromatograph (Hongtu-sp7890, Shandong Lunan Ruihong Analytical Instrument Co. Ltd) is used to examine amount of hydrogen and oxygen during testing process. Faraday efficiency can be calculated by the following formula:

$$FE_{O_2} = \frac{4 \times n_{O_2} \times 10^{-3}}{\frac{Q}{96500}} \times 100\%$$

$$FE_{H_2} = \frac{2 \times n_{H_2} \times 10^{-3}}{\frac{Q}{96500}} \times 100\%$$

Q is the total charge passed (108 C), n_{O_2} is molar mass of oxygen and n_{H_2} is molar mass hydrogen.

ES3. DFT Calculations

DFT calculations were performed using the VASP with the Perdew−Burke−Ernzerhof (PBE) level and project-augmented wave (PAW) method. The project-augmented wave (PAW) method was used to represent the core−valence electron interaction. A 4×4×1 Monkhorst-Pack grid of k-points was used for the Brillouin zone integration. The valence electronic states were expanded in plane-wave basis sets with an energy cutoff at 570 eV. Gaussian smearing of 0.05 eV was applied during the geometry optimization. The convergence criteria for the iteration in the self-consistent field (SCF) were set at 10^{-6} eV, and the residual force for optimizing atom positions was less than 0.02 eV/Å.

Supplementary Figures S1-S34

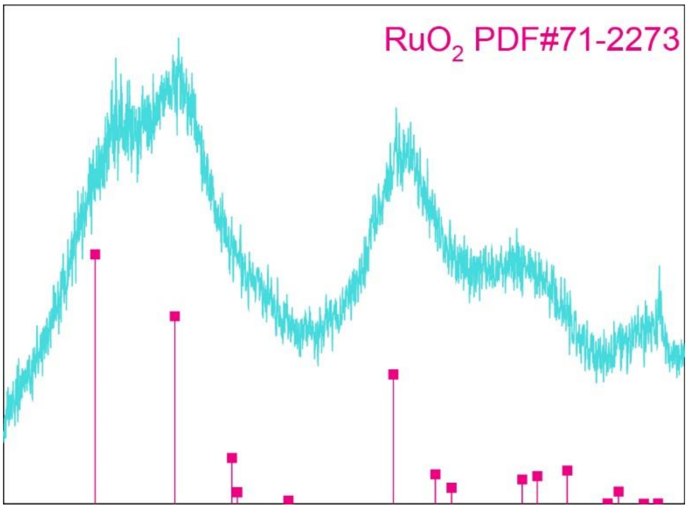

Figure S1. The XRD pattern of RuO_2-200, which shows that the XRD peaks are broad, demonstrating the size of RuO_2-200 is small and the crystalline is poor.

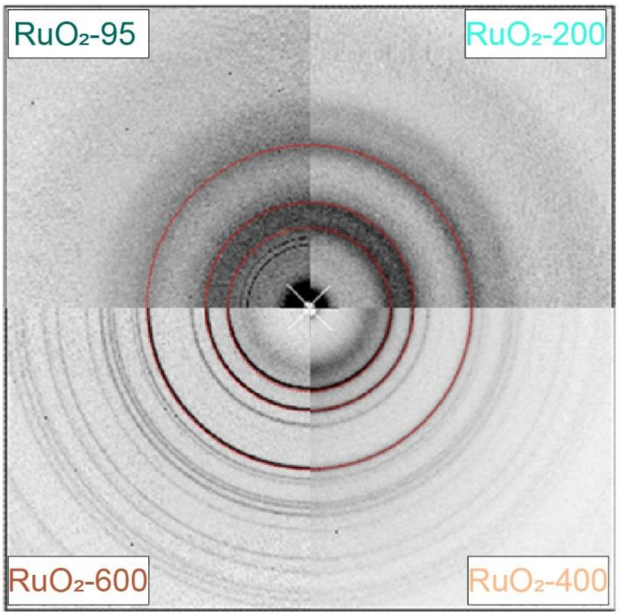

Figure S2. The Synchrotron X-ray diffraction patterns of RuO_2-95, RuO_2-200, RuO_2-400, and RuO_2-600 catalysts.

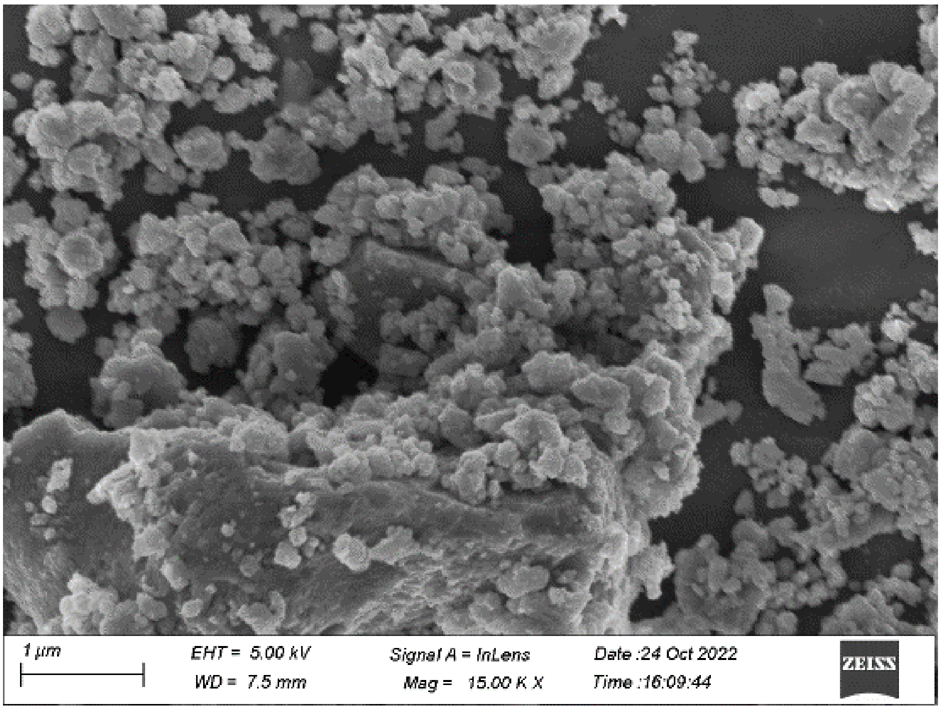

Figure S3. The SEM image of RuO_2-95.

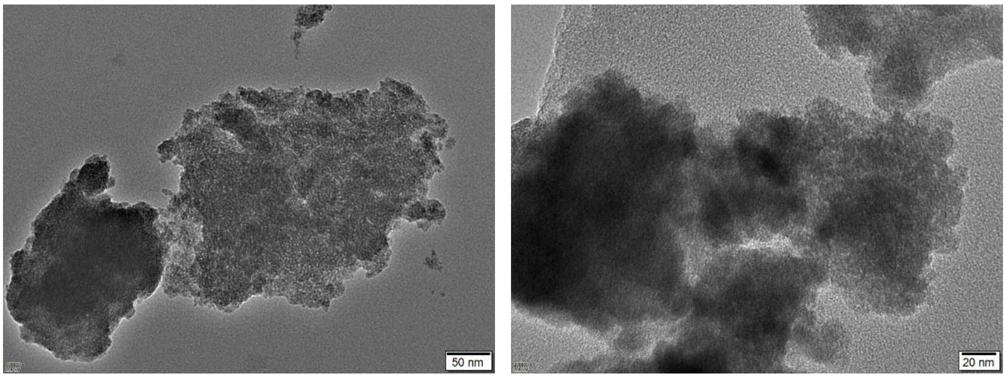

Figure S4. The TEM images of RuO_2-95.

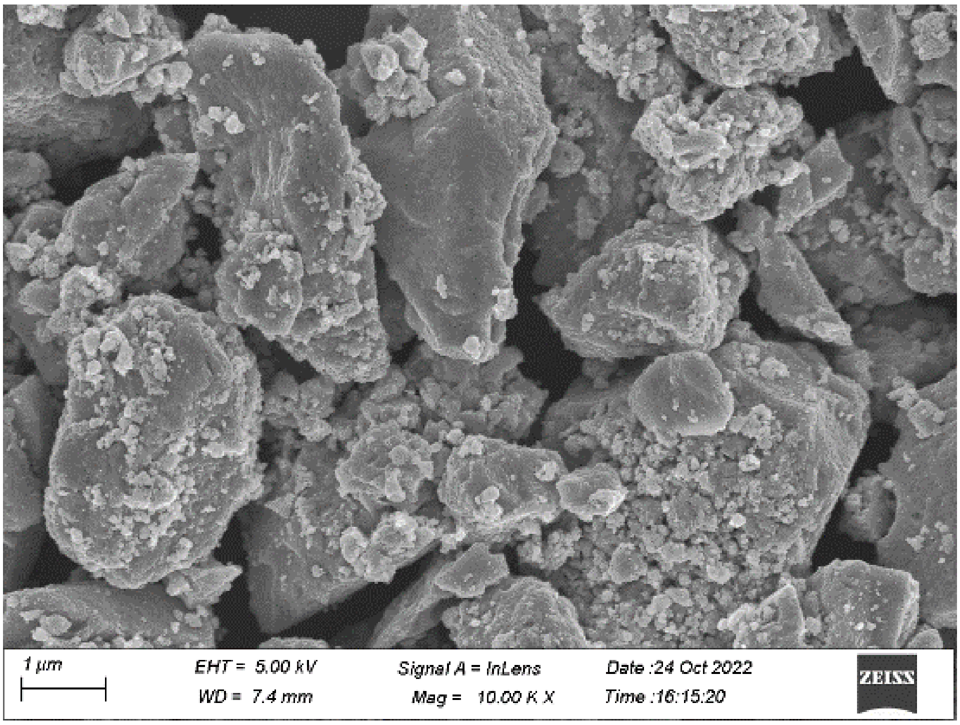

Figure S5. The SEM image of RuO_2-200.

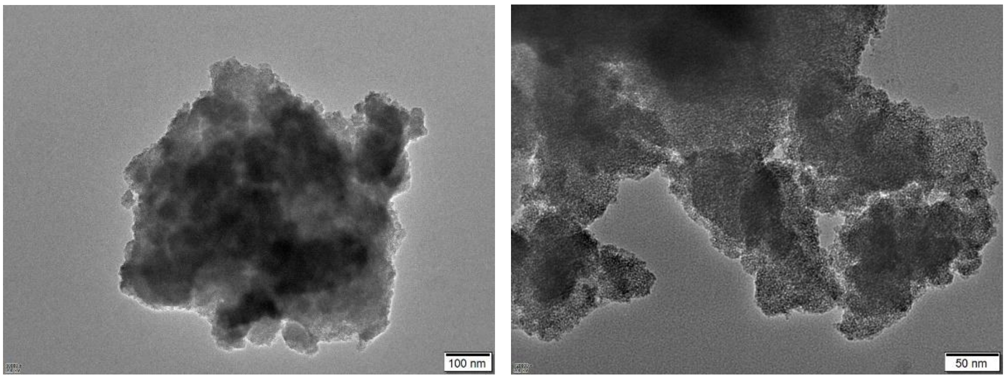

Figure S6. The TEM images of RuO_2-200.

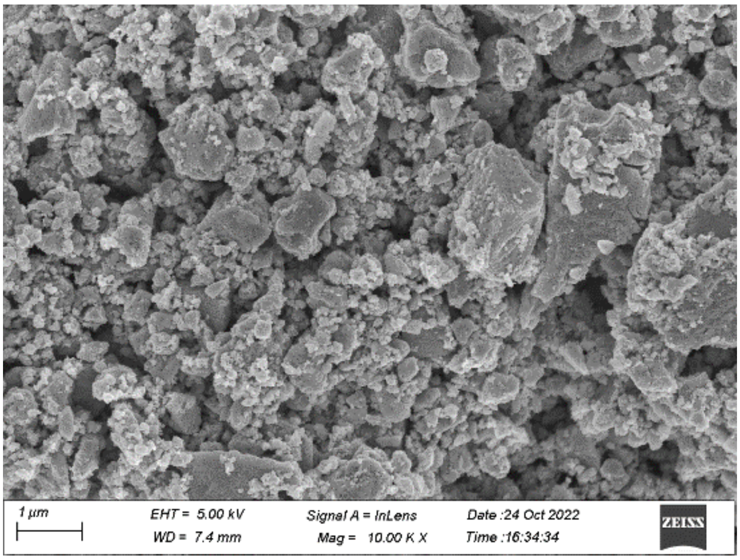

Figure S7. The SEM image of RuO_2-400.

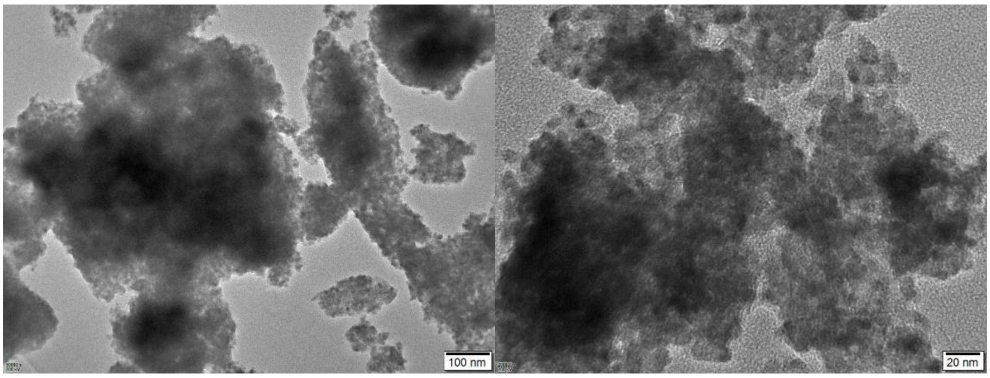

Figure S8. The TEM images of RuO_2-400.

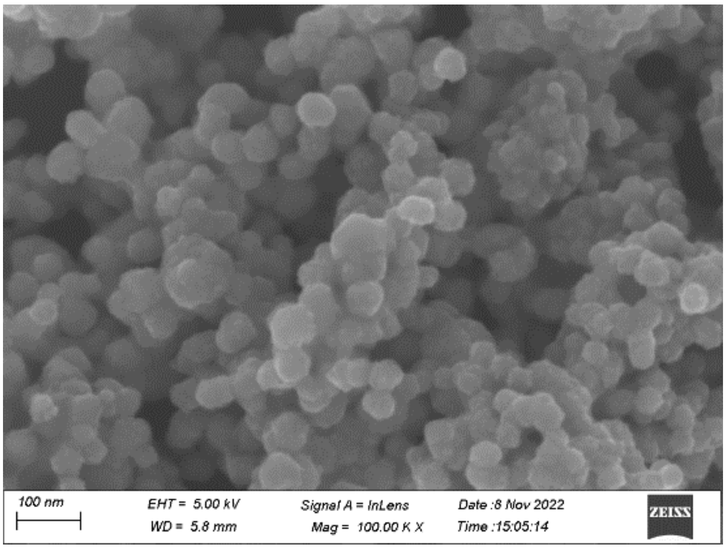

Figure S9. The SEM image of RuO_2-600.

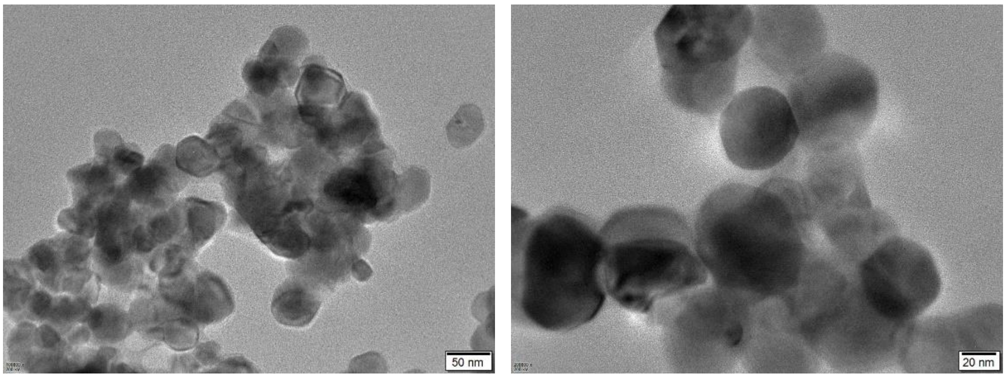

Figure S10. The TEM images of RuO_2-600.

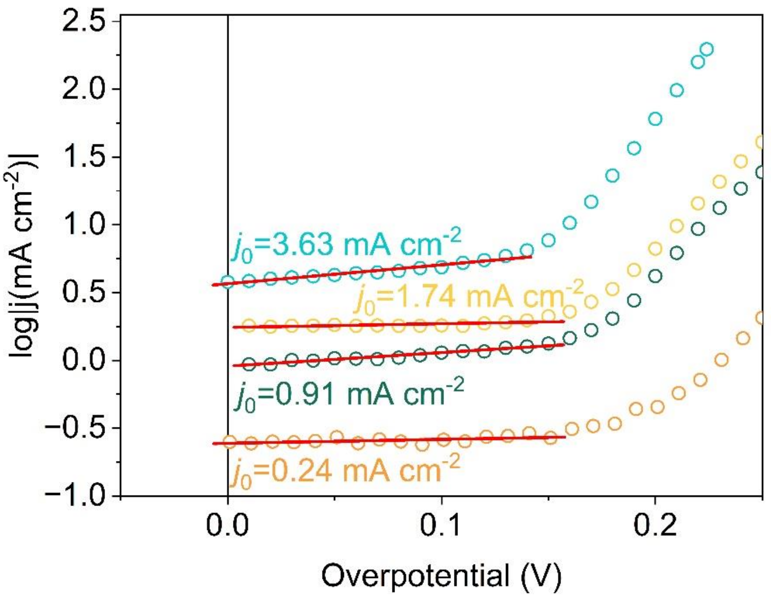

Figure S11. The exchange current density for OER on the as-prepared RuO_2 catalysts.

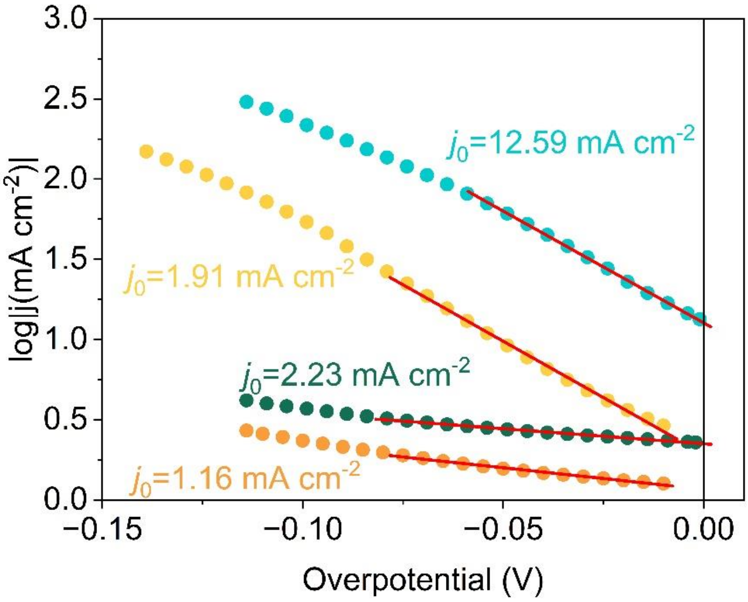

Figure S12. The exchange current density for HER on the as-prepared RuO_2 catalysts.

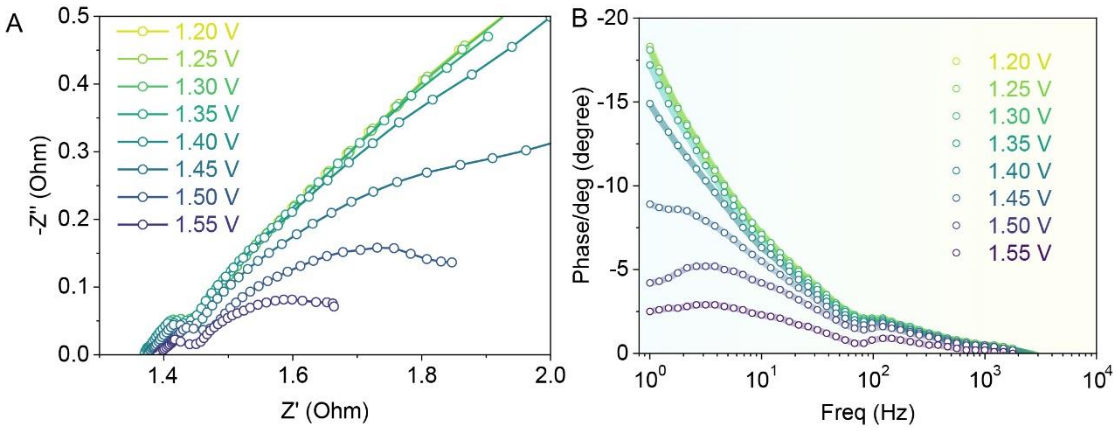

Figure S13. The EIS of OER on RuO_2-200 at different potentials. (A) Nyquist plots and (B)the bode plots.

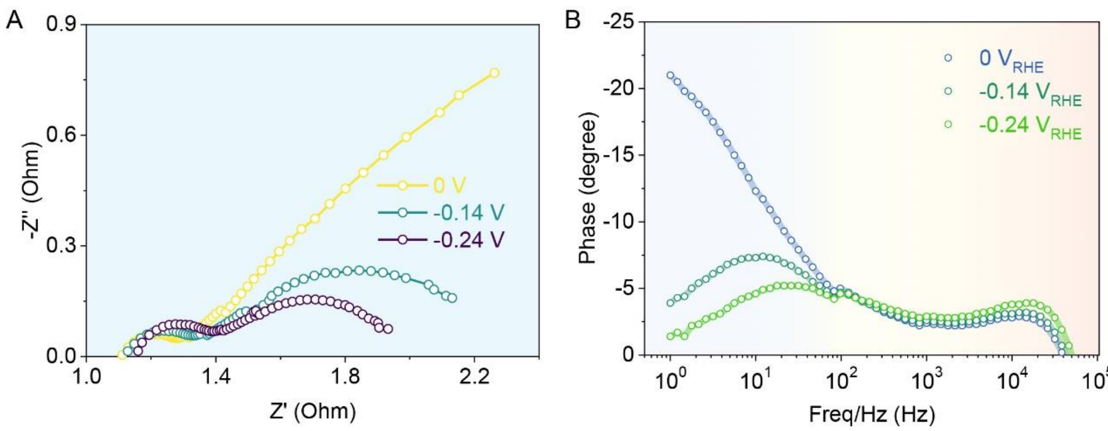

Figure S14. The EIS of HER on RuO_2-200 at different potentials. (A) Nyquist plots and (B)the bode plots..

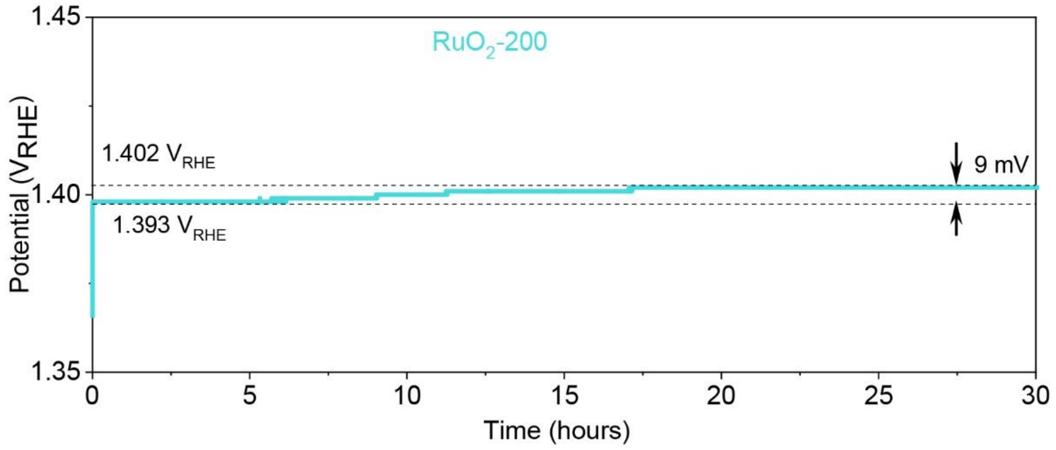

Figure S15. The chronopotentiometry test on RuO_2-200 at 10 mA·cm^{-2} of current density for 30 hours.

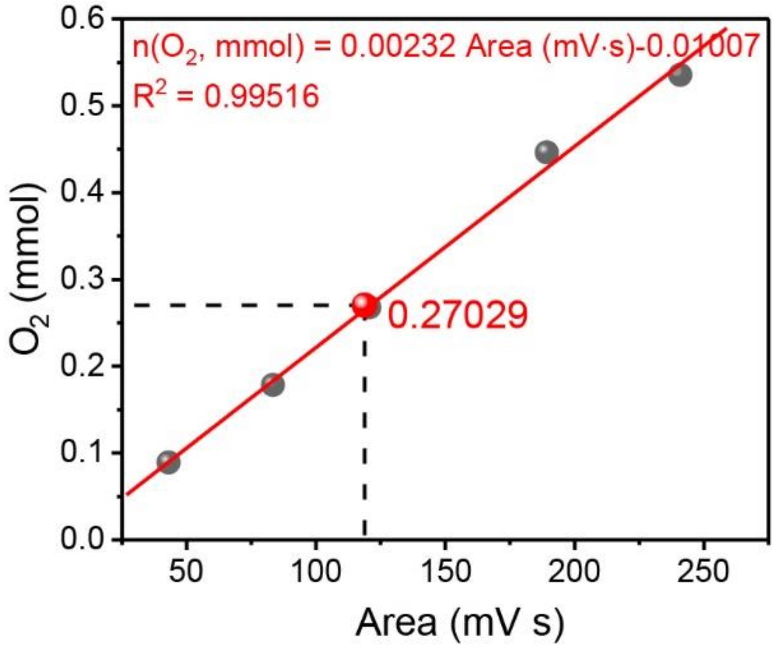

Figure S16. The produced O_2 at 10 mA cm^{-2} of current density for 3 hours, the generated gas was measured by a gas chromatography. Under the current density, the generated rate of O_2 was calculated 0.09 mmol h^{-1}.

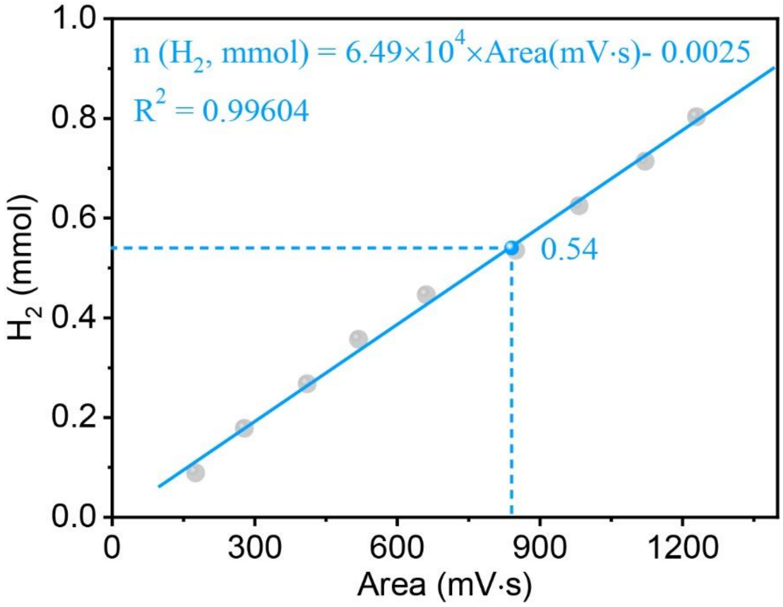

Figure S17. The produced H_2 at 10 mA cm^{-2} of current density for 3 hours, the generated gas was measured by a gas chromatography. Under the current density, the generated rate of O_2 was calculated 0.18 mmol h^{-1}.

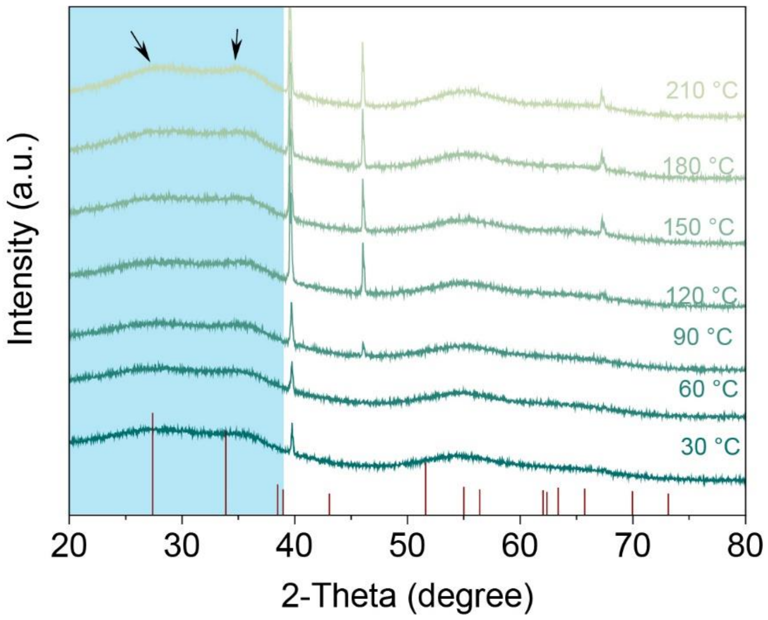

Figure S18. The in-situ XRD patterns of RuO_2 from 30ºC to 210ºC.

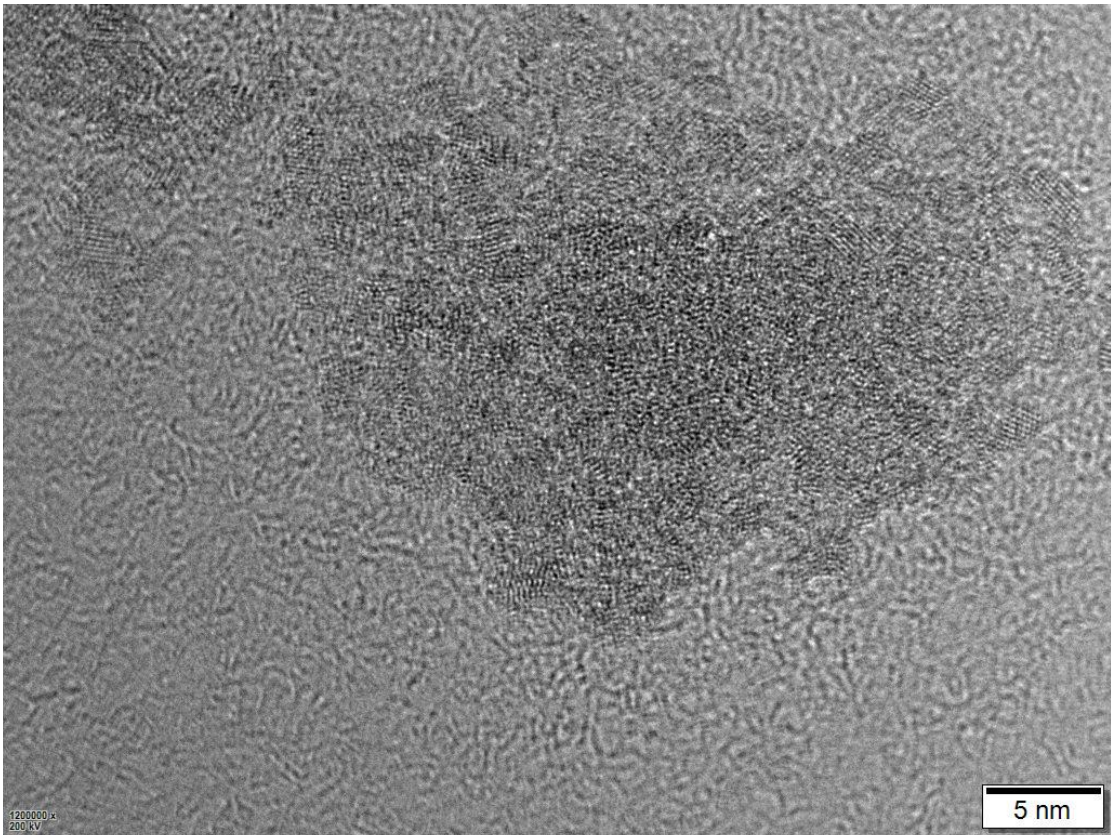

Figure S19. The HRTEM image of RuO_2-95.

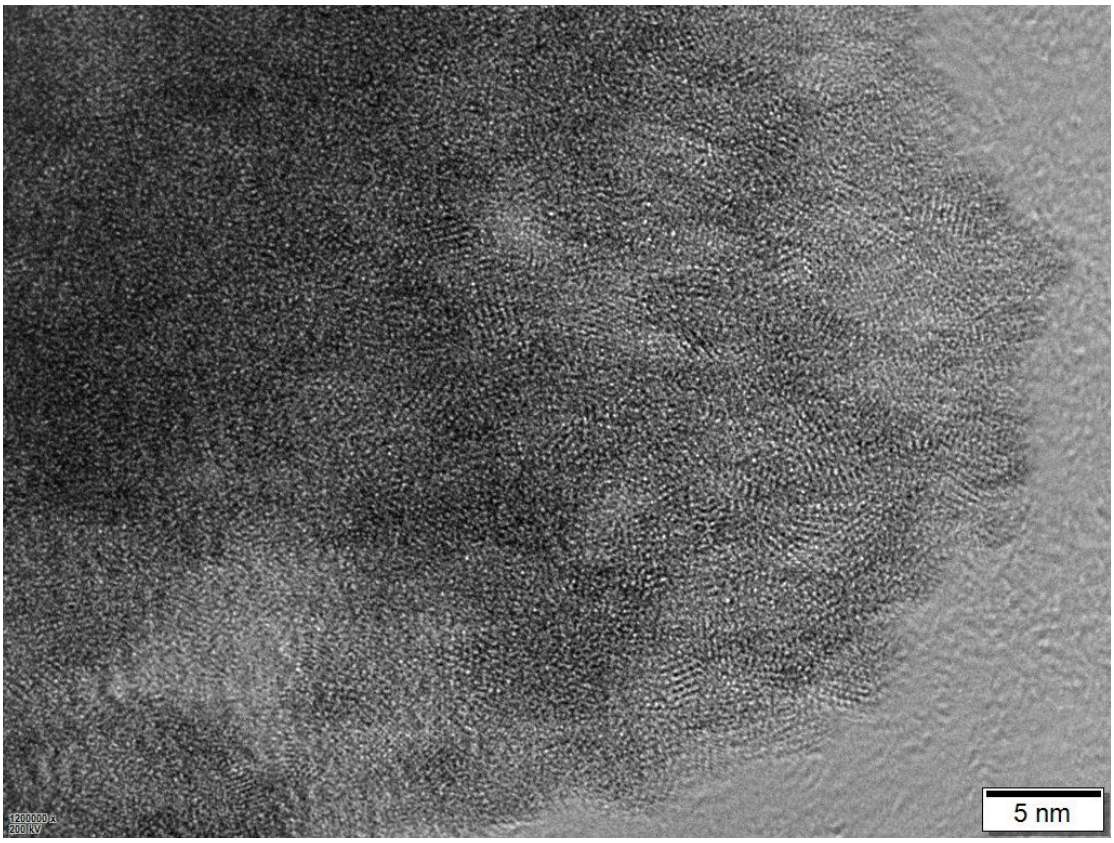

Figure S20. The HRTEM image of RuO_2-200.

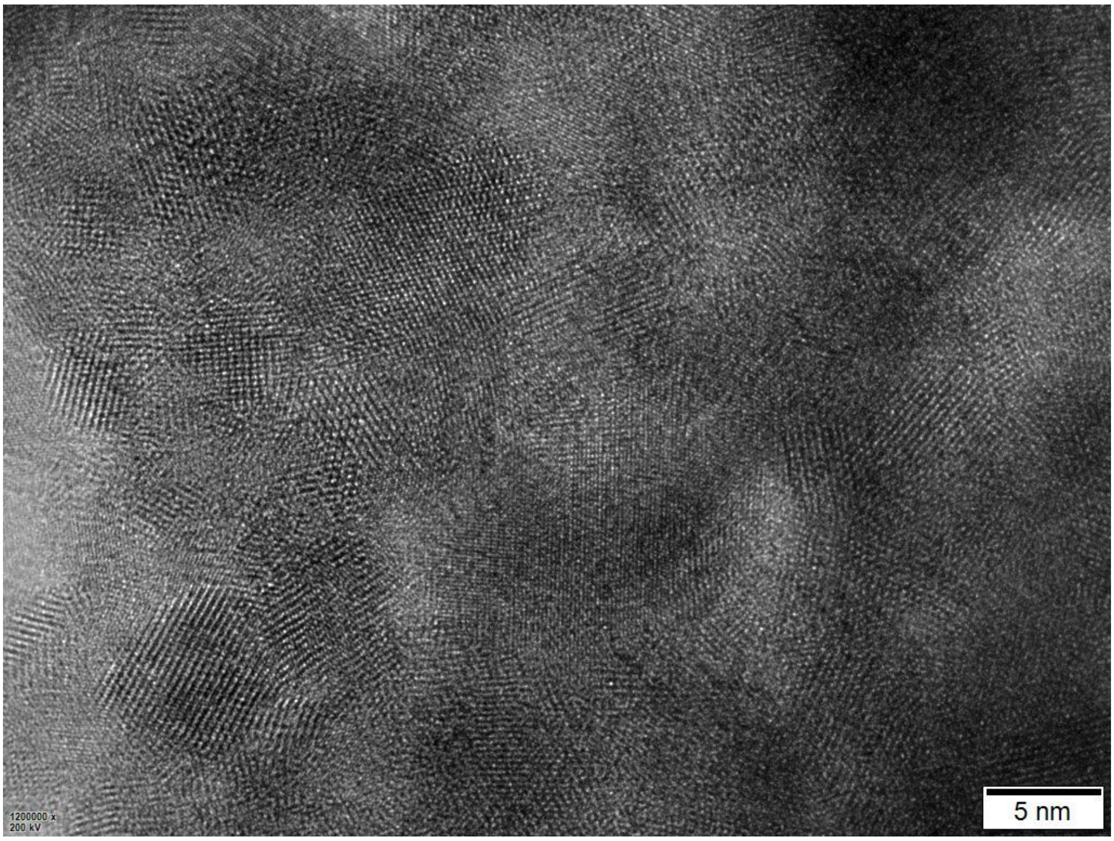

Figure S21. The HRTEM image of RuO_2-400.

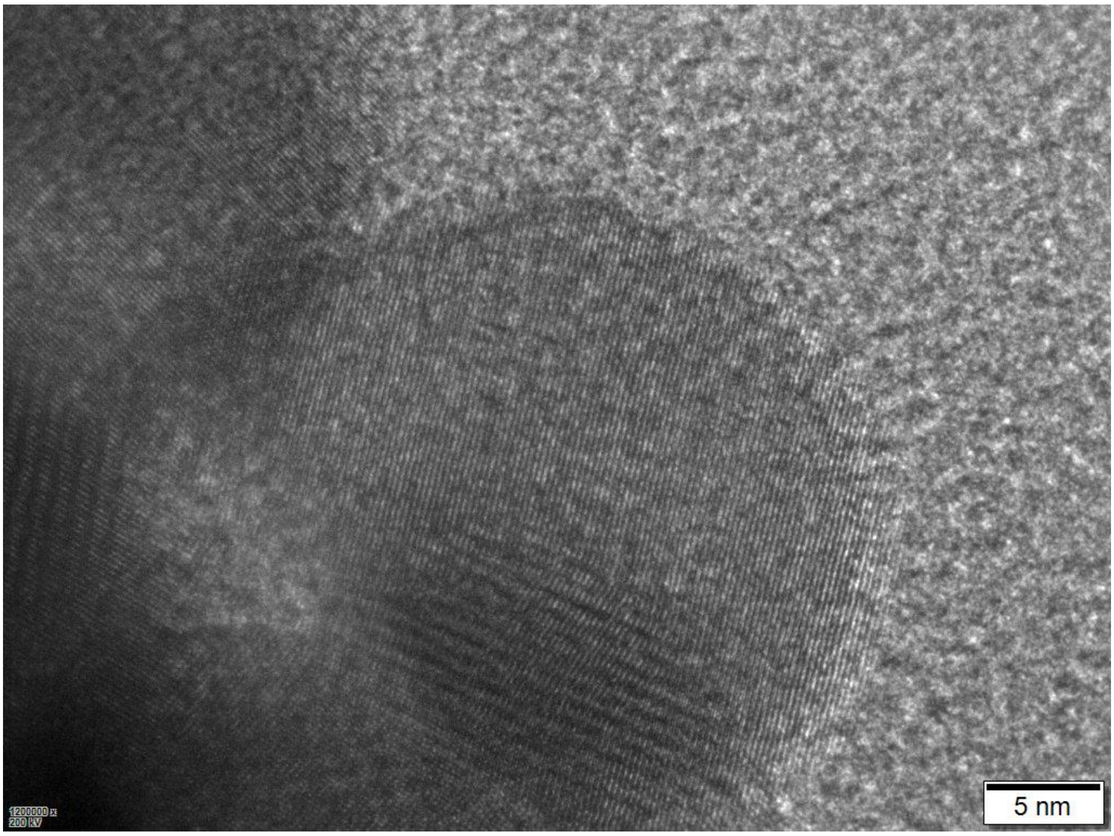

Figure S22. The HRTEM image of RuO_2-600.

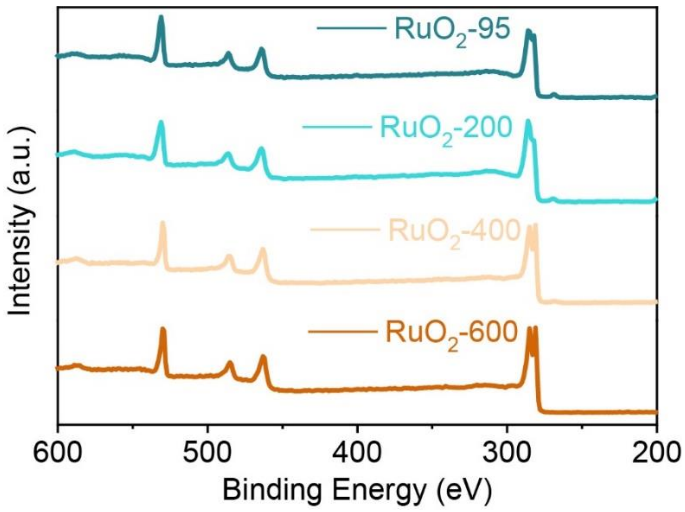

Figure S23. The survey XPS of series of RuO_2 clusters or nanocrystals.

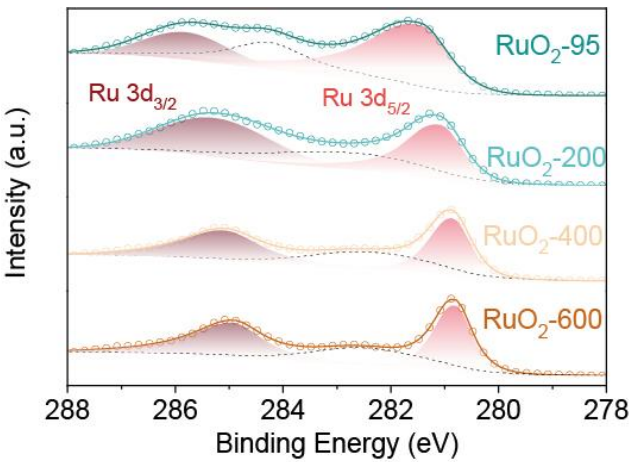

Figure S24. The high resolution XPS of Ru 3d (the dash line is the C 1s and Ru 3d satellite peaks).

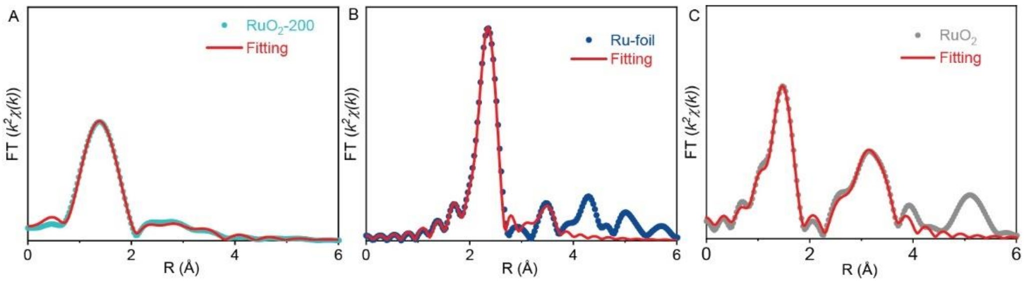

Figure S25. The Ru K EXAFS fitting of (A) RuO_2-200, (B) Ru foil and (C) RuO_2.

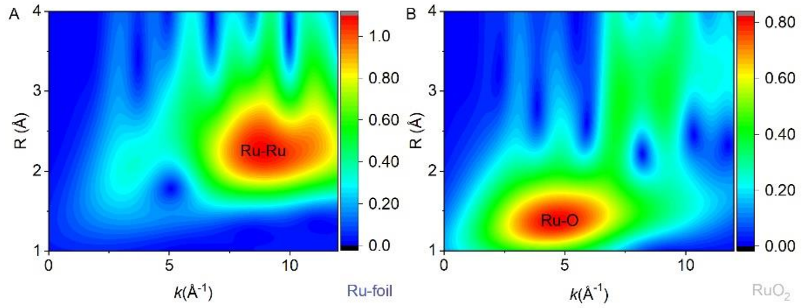

Figure S26. The wavelet transform-EXAFS of the (A) Ru foil and (B) RuO_2.

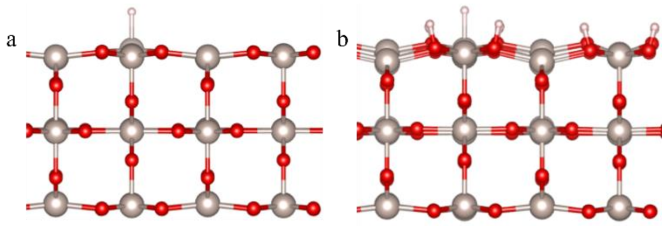

Figure S27. The geometric configuration of (A) *H-RuO_2; (B) *H-RuO_2-OH.

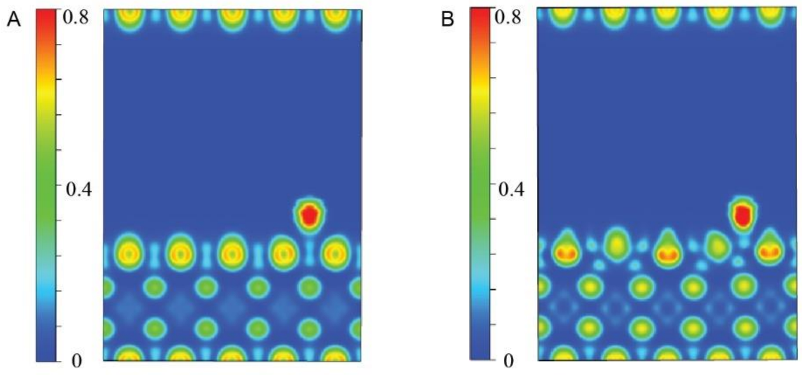

Figure S28. The Electron localization function (ELF) of (A) *H-RuO_2; (B) *H-RuO_2-OH.

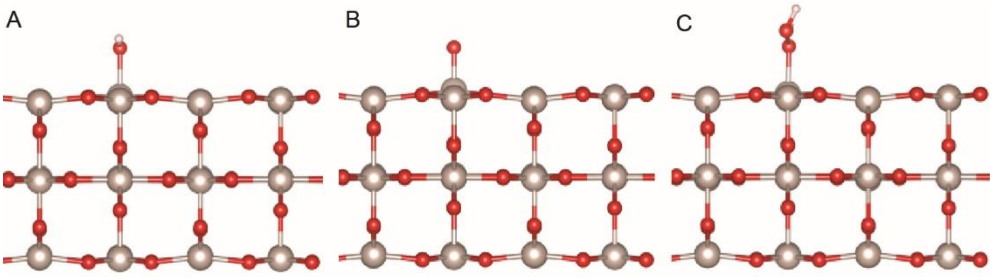

Figure S29. The geometric configuration of (A) *OH-RuO_2; (B) *O-RuO_2, (C)*OOH-RuO_2.

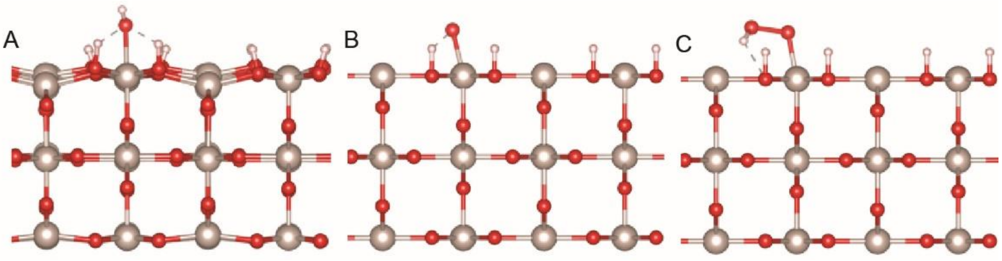

Figure S30. The geometric configuration of (a) *OH-RuO_2-OH; (b) *O-RuO_2-OH, (c)*OOH-RuO_2-OH.

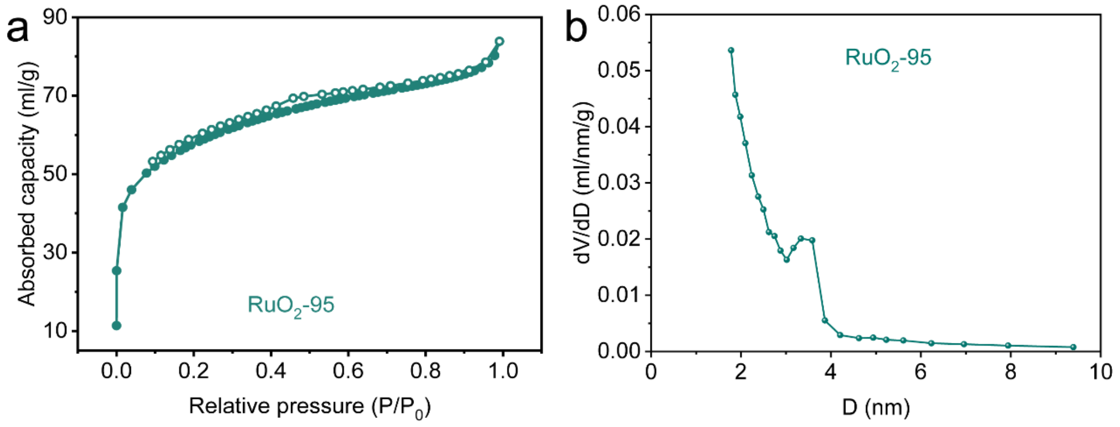

Figure S31. (a) the adsorption-desorption curve of the RuO_2-95 and (b)the pore size dispersing.

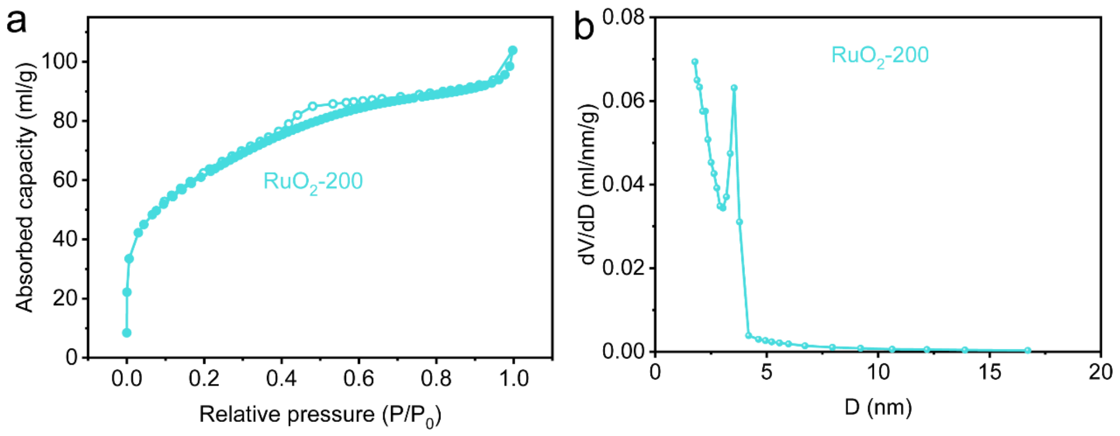

Figure S32. (a) the adsorption-desorption curve of the RuO_2-200 and (b)the pore size dispersing.

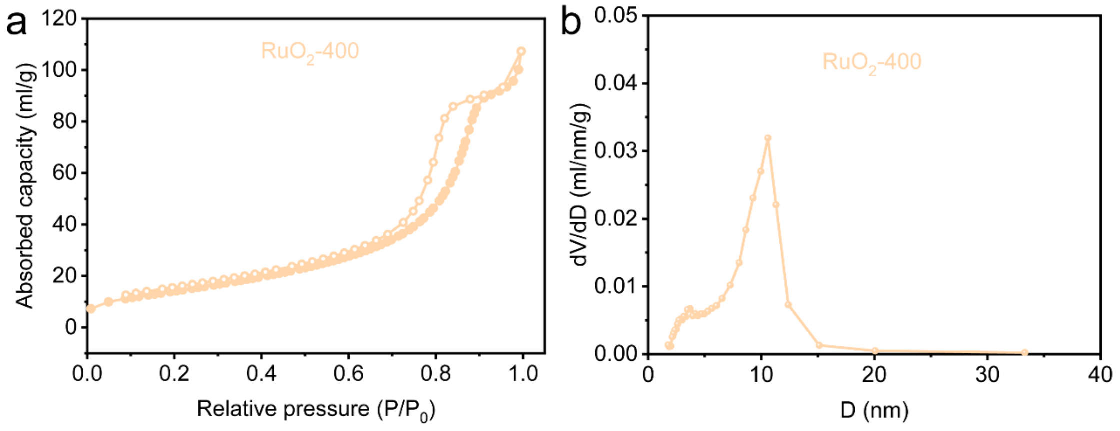

Figure S33. (a) the adsorption-desorption curve of the RuO_2-400 and (b)the pore size dispersing.

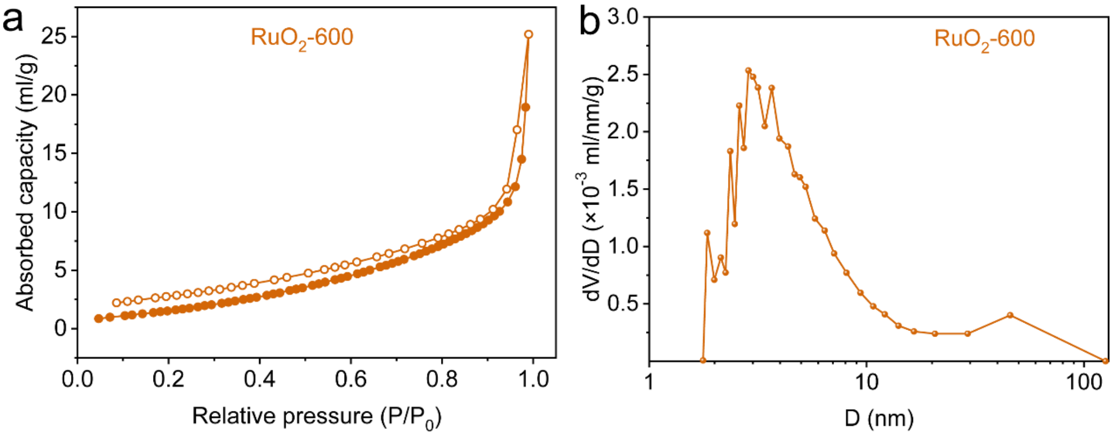

Figure S34. (a) the adsorption-desorption curve of the RuO_2-600 and (b)the pore size dispersing.

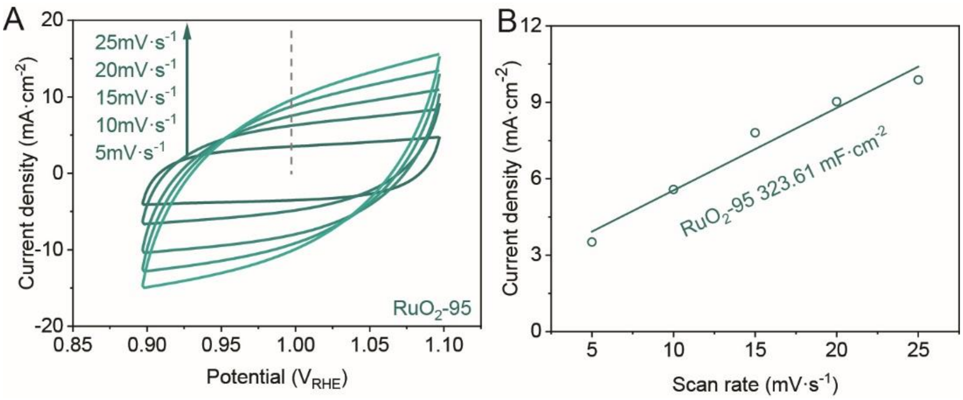

Figure S35. (A) The cyclic voltammogram curve on RuO_2-95 with series of scanning speed and (B) the calculated C_{dl}.

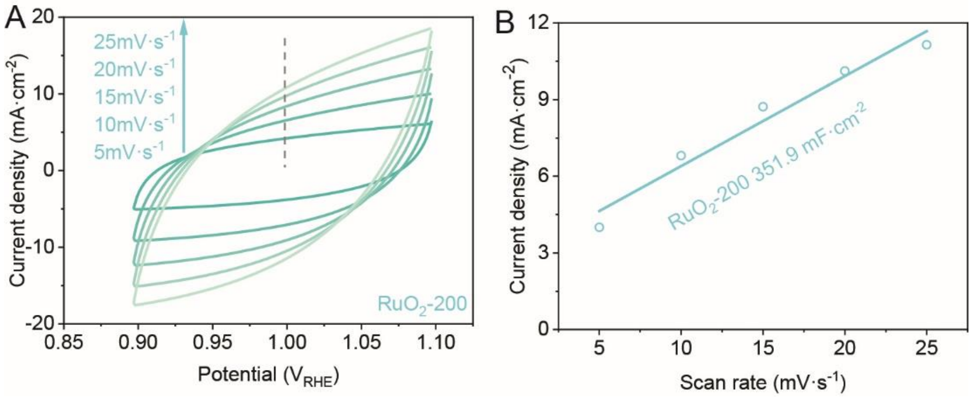

Figure S36. (A) The cyclic voltammogram curve on RuO_2-200 with series of scanning speed and (B) the calculated C_{dl}.

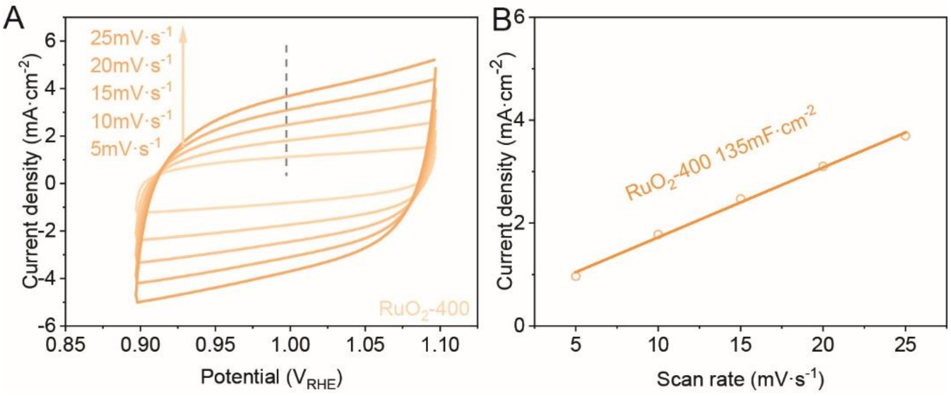

Figure S37. (A) The cyclic voltammogram curve on RuO_2-400 with series of scanning speed and (B) the calculated C_{dl}.

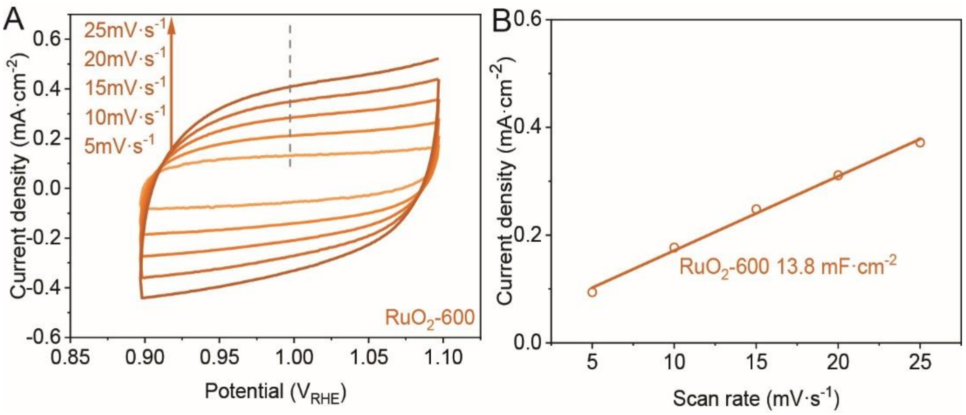

Figure S38. (A) The cyclic voltammogram curve on RuO_2-600 with series of scanning speed and (B) the calculated C_{dl}.

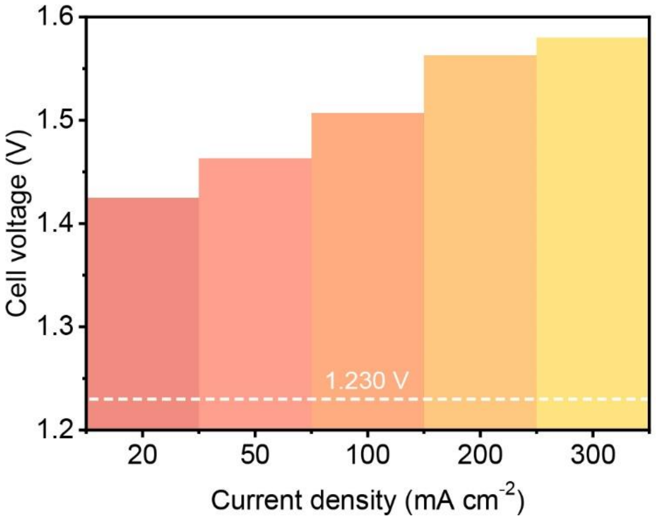

Figure S39. The cell voltages for water splitting depending on current density, calculated from the polarized curves on HER and OER.

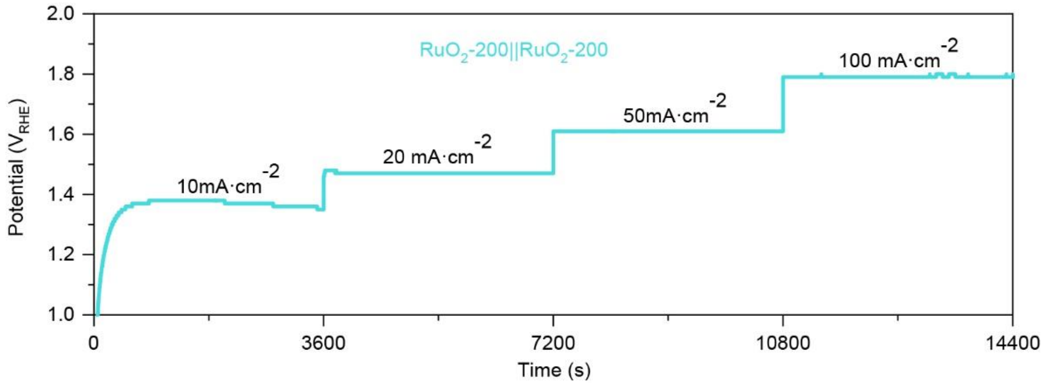

Figure S40. The Chronopotentiometry test at a series of current densities, which shows that the RuO_2-200 performs a high stability under different current densities.

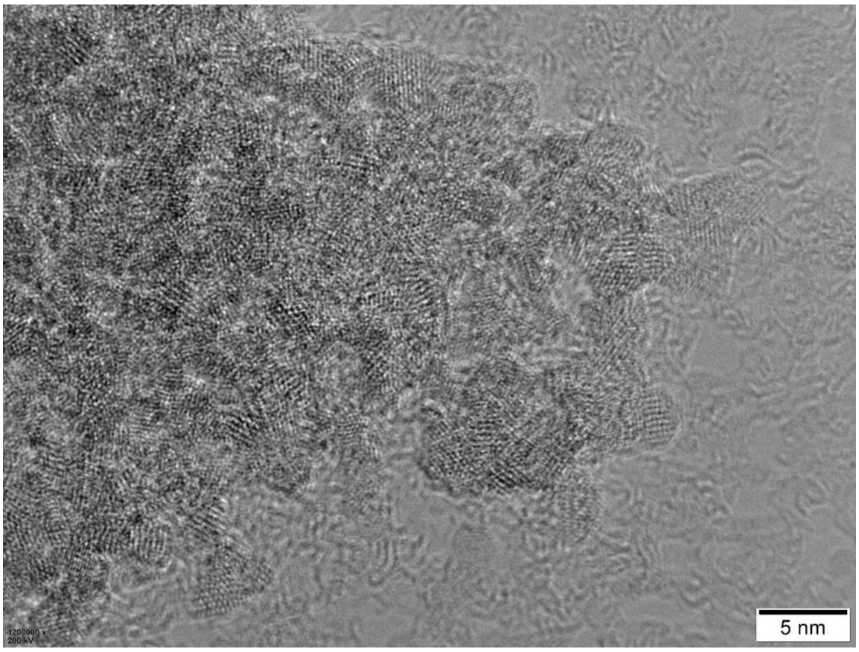

Figure S41. The HRTEM image of RuO_2-200 after OER test.

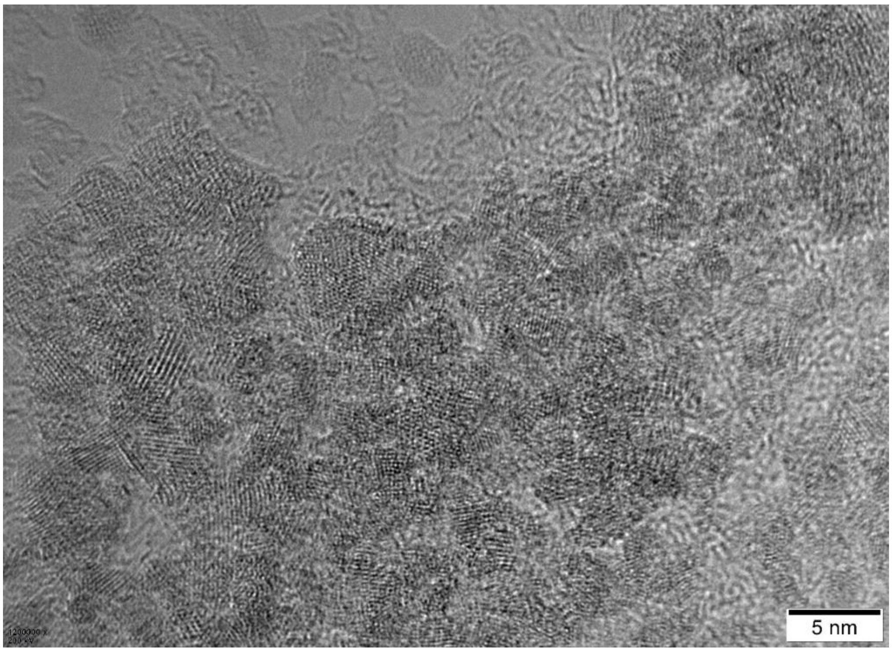

Figure S42. The HRTEM image of RuO_2-200 after HER test.

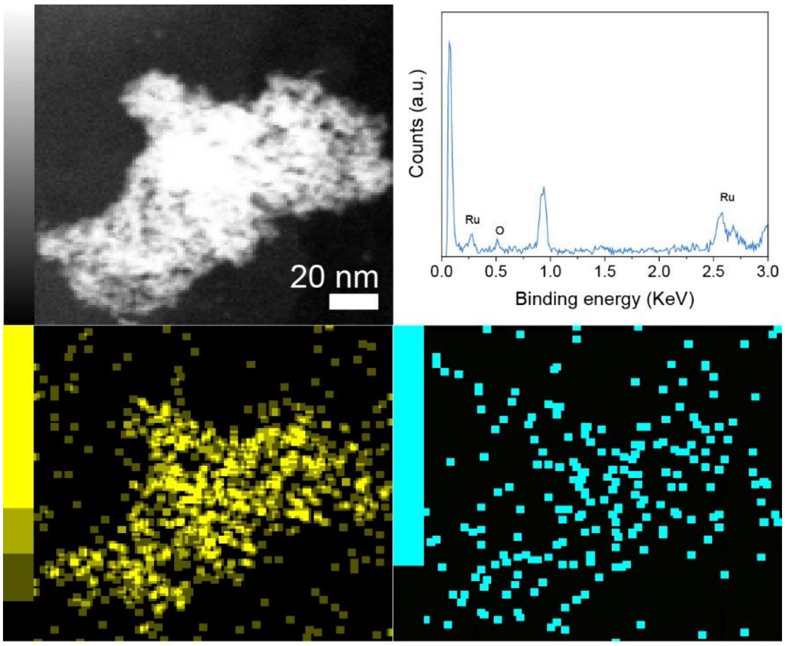

Figure S43. The HAADF-STEM image and Element analysis of the RuO_2-200 after OER test.

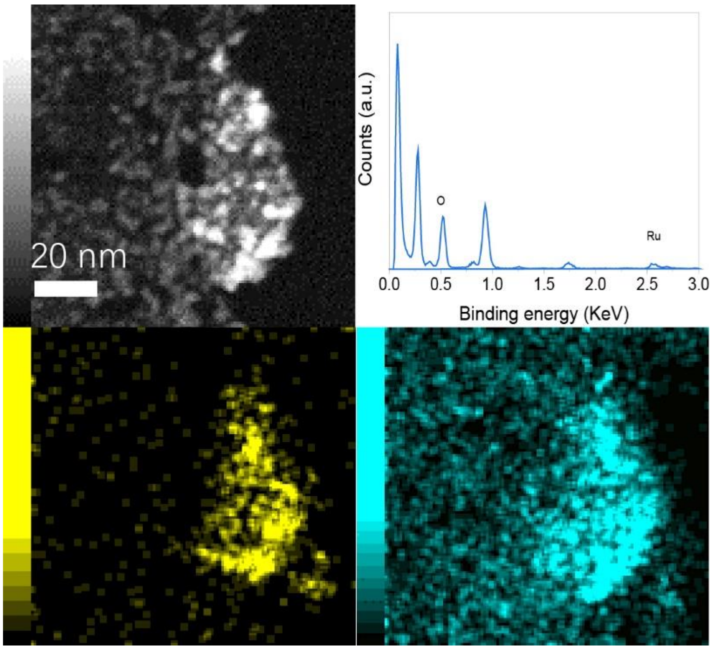

Figure S44. The HAADF image and Element analysis of the RuO_2-200 after HER.

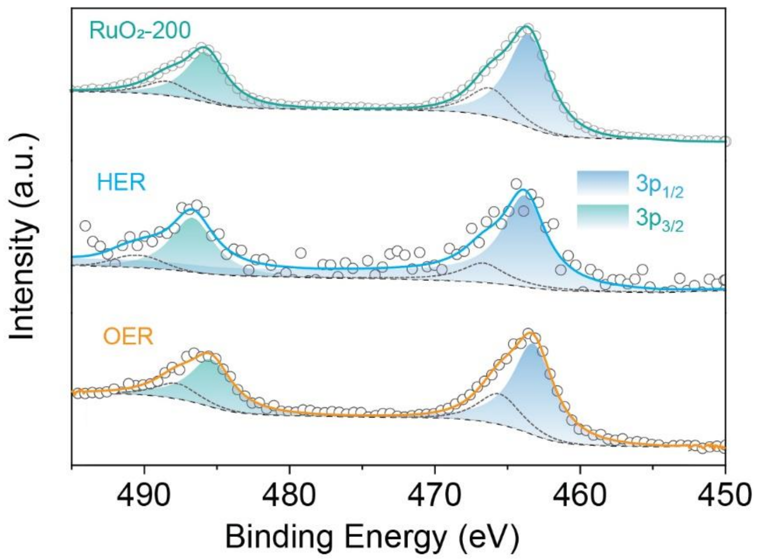

Figure S45. The Ru 3p XPS comparison of RuO_2-200 before and after HER/OER test.

Supplementary Tables S1-S5

Table S1. The locations of the Ru 3d and 3p XPS peaks in RuO_2.

	$3d_{3/2}$	$3d_{5/2}$	$3p_{1/2}$	$3p_{3/2}$
RuO_2-95	284.32	281.17	462.85	484.94
RuO_2-200	285.17	281.06	462.59	484.88
RuO_2-400	285.13	280.87	462.36	484.73
RuO_2-600	284.89	280.83	462.36	484.7

Note: The FWHMs of $3d_{3/2}$ and $3d_{5/2}$ were set as 1.32 and 0.88 respectively, while that of $3p_{1/2}$ and $3p_{3/2}$ were set as 2.5.

Table S2. The locations of the O1s XPS peaks in RuO_2.

	H_2O	OH^-	O^{2-}
RuO_2-95	532.53	531.34	529.63
RuO_2-200	532.01	530.24	529.48
RuO_2-400	-	530.43	529.44
RuO_2-600	-	530.54	529.40

Note: The O 1s FWHMs of OH^- and O^{2-} were set as 1.50 and 0.95.

Table S3. The O ratios of area integral intensity calculated by the corresponding XPS peaks.

	Area of the XPS peaks			Ratio of area integral intensity			OH^-/O^{2-}
	H_2O	OH^-	O^{2-}	H_2O	OH^-	O^{2-}	
RuO_2-95	80206.03	32709.28	16534.02	61.96%	25.27%	12.77%	1.98
RuO_2-200	77065.59	51032.18	24460.03	50.52%	33.45%	16.03%	2.09
RuO_2-400	0	87152.56	56633.35	0.00%	60.61%	39.39%	1.54
RuO_2-600	0	80316.92	71893.79	0.00%	52.77%	47.23%	1.12

Table S4. The fitting results of Ru k-edge EXAFS of RuO_2-200 and Ru foil.

Sample	bond type	CN*	R (Å)	σ^2 (10^{-3}Å^2)**	R factor
RuO_2-200	Ru-O1	1.9±0.2	1.91±0.02	4.4±2.4	0.014
	Ru–O2	3.8±0.1	2.04± 0.01	2.9±2.1	
Ru-foil	Ru-Ru	12	2.66±0.01	3.7±1.2	0.006

* CN: coordination number; S02 was fixed to be 0.88 from Ru foil.

** σ2: Debye−Waller factors

Table S5. BET results of series of RuO_2 catalysts.

**Sample**	**RuO_2-95**	**RuO_2-200**	**RuO_2-400**	**RuO_2-600**
a_s, BET [$m^2\ g^{-1}$]	184.1	214.6	53.1	7.2
Pore size (nm)	3.19	3.07	11.32	8.05

Table S6. Comparisons of the stabilities of Ru-based catalysts and RuO_2-200 for water splitting and the overpotentials at 10 mA cm^{-2} on HER/OER.

Catalysts	Stability time/ h@ conditions	$\eta_{@10mA\ cm^{-2}}$ for HER/ mV	$\eta_{@10mA\ cm^{-2}}$ for OER/ mV	Reference
RuO_2-200	100@1.5V	42@50 mA cm^{-2}	199@50 mA cm^{-2}	This work
RuIr-NC	120@10 mA cm^{-2}	46	165	*Nat. Commun. 2021, 12 (1), 1145.*
RuO_2 NW	65@10 mA cm^{-2}	30	234	*Adv. Func. Mater. 2018, 28 (41).*
Ru–$NiSe_2$/NF*	20@10 mA cm^{-2}	29	210	*Small 2022, 18 (6), e2105305.*
SA-Ru /$Ti_3C_2T_x$*	32@1.5 V	70	290	*Small 2020, 16 (33), e2002888*
RuCu NSs*	12@10 mA cm^{-2} (OER)	20	234	*Angew. Chem. Int. Ed. 2019, 58 (39), 13983-13988.*
Ru@V-RuO_2/C	25@10 mA cm^{-2}	46	176	*Adv. Mater. 2023, e2206351.*
RuO_2-WC NPs*	10@10 mA cm^{-2}	58	347	*Angew. Chem. Int. Ed.* 2022, *61* (21), e202202519.
Ru@Ni-B	72@10 mA cm^{-2}	14	180	*Adv. Sustain. Syst. 2021, 5 (8), 202000184.*
Ru_3Ni_3 Nas	10@10 mA cm^{-2}	39	252	*iScience 2019, 11, 492-504.*
M-$ZnRuO_x$	20@20 mA cm^{-2}	17	224	*Small 2023, 19, 2207235.*
Br-Ru-RuO_2/MCC	12@10 mA cm^{-2}	77	300	J Colloid Interface Sci. 2023, 644, 238–245.

Catalysts	Stability time/ h@ conditions	$\eta_{@10mA\ cm^{-2}}$ for HER/ mV	$\eta_{@10mA\ cm^{-2}}$ for OER/ mV	Reference
NF@NiO@Ru*	24@1.55 V	51	226	*ChemPhysChem 2021, 22, 1 –8*
Ru@$MoO(S)_3$	24@10 mA cm^{-2}	63	226	*Nano Energy 2022, 100., 107445*

*SA: Single atomic, NPs: nanoparticles, NCs: nanocrystals, NF: Ni foam.
